# Supplementary material for: Associations between inflexible job conditions, health and healthcare utilisation in England: retrospective cross-sectional study
Source: BMJ Open. 2022 Dec 5;12(12):e062942. doi: 10.1136/bmjopen-2022-062942 (PMC9723827; doi:10.1136/bmjopen-2022-062942)
Supplement: Supplementary data [file bmjopen-2022-062942supp001.pdf]

SUPPLEMENTARY MATERIAL

Regression equations

1. The effect of having an inflexible job on EQ-5D utility score and EQ-5D domains:

$$H_{ijl} = \beta.inflexible_{ijl} + \boldsymbol{\gamma_1 X_{ij}} + \tau_l + \delta_t + \varepsilon_{ijl} \quad (1)$$

Where *H* are the health outcomes of individual *i* living in LSOA *j* in local authority *l*;

inflexible is an indicator equal to one if an individual reports that their job is inflexible;

X_{ij} is a vector of covariates at individual and LSOA level including indicators for gender, ten-year age band, index of multiple deprivation decile, parental status, ethnicity, and whether the respondent ever smoked;

τ_l are LAD fixed-effects;

δ_t are time fixed-effects, and ε_{ijl} is an error term.

2. The effect of having an inflexible job on the length of time since the respondent last saw a GP or nurse, and use of out-of-hours (healthcare utilisation):

$$HCU_{ijlp} = \beta.inflexible_{ijl} + \boldsymbol{\gamma_1 X_{ij}} + \boldsymbol{\gamma_2 LTC_i} + \gamma_3.availability_p + \tau_l + \delta_t + \varepsilon_{ijl} \quad (2)$$

Where *HCU* are the healthcare utilisation outcomes of individual *i* living in LSOA *j* in local authority *l* registered at general practice *p*;

LTC_i is a vector of indicators for whether individual *i* has each long-term condition;

$availability_p$ is a measure of the average appointment availability at practice *p*;

inflexible, X_{ij}, τ_l, δ_t and ε_{ijl} are as in equation (1).

3. The mediating effect of health care use in the relationship between having an inflexible job and health:

Moss C, *et al. BMJ Open* 2022; 12:e062942. doi: 10.1136/bmjopen-2022-062942

$$HCU_{ijl} = \beta.inflexible_{ijl} + \boldsymbol{\gamma_1 X_{ij}} + \tau_l + \delta_t + \varepsilon_{ijl} \quad (3)$$

$$H_{ijl} = \beta.\widehat{HCU}_{ijl} + \boldsymbol{\gamma_1 X_{ij}} + \tau_l + \delta_t + u_{ijl} \quad (4)$$

Two-stage least squares where equation (3) is the first stage and (4) is the second stage.

Standard Occupational Classifications (SOCs)

At the LAD level, we obtained annual information on the share of the working population employed in each of the nine broad categories of Standard Occupational Classification (SOC)[29]: 1) managers, directors and senior officials; 2) professional; 3) associate professional and technical; 4) administrative and secretarial; 5) skilled trade; 6) caring, leisure and other service; 7) sales and customer services; 8) process, plant and machine operatives; and 9) elementary occupations. We obtained these variables via NOMIS: the UK Labour Market statistics portal [30] and merged them by LAD to the individual-level data.

We intended to include the percentage of the population employed in each sector as a covariate to adjust for other job characteristics. However when included, they were not jointly statistically significant, and their inclusion did not alter the coefficient on the inflexible job indicator.

Moss C, *et al. BMJ Open* 2022; 12:e062942. doi: 10.1136/bmjopen-2022-062942

Supplementary Table 1: Possible responses to survey questions "when did you last see or speak to a GP [nurse] from your GP surgery?" and corresponding intervals in main analysis; and value assigned in two-stage least squares regressions

Response	Months since last saw/spoke to GP/nurse	Value in two-stage least squares regressions
In the past 3 months	0 to 3	1
Between 3 and 6 months ago	3 to 6	2
Between 6 and 12 months ago	6 to 12	3
More than 12 months ago	12 to 24	4
I have never seen a GP from my GP surgery	24+	5

Data source: General Practice Patient Survey, Questions 1 and 2

Moss C, *et al. BMJ Open* 2022; 12:e062942. doi: 10.1136/bmjopen-2022-062942

Supplementary Table 2: Local Authority Districts with lowest percentage of respondents reporting an inflexible job

Local Authority District	% reporting inflexible job	N
Isles of Scilly	13.1	175
City of London	19.2	240
Cambridge	19.4	2,847
Ryedale	20.7	1,282
Oxford	21.0	4,000
Melton	21.0	932
South Lakeland	21.3	2,987
South Cambridgeshire	21.8	4,509
West Somerset	21.9	987
Vale of White Horse	22.1	2,676
Cotswold	22.3	2,312
Rutland	22.4	706
Wokingham	22.8	3,156
South Oxfordshire	22.9	3,039
South Somerset	22.9	4,517
Stroud	22.9	3,377
Derbyshire Dales	23.1	1,969
West Oxfordshire	23.2	2,498
Wiltshire	23.3	10,662
Eden	23.3	1,813

Data source: GPPS respondents in full-time employment, 2012-2017

Moss C, *et al. BMJ Open* 2022; 12:e062942. doi: 10.1136/bmjopen-2022-062942

Supplementary Table 3: Local Authority Districts with highest percentage of respondents reporting an inflexible job

Local Authority District	% reporting inflexible job	N
Barking and Dagenham	40.7	6,772
Newham	38.3	9,043
Knowsley	37.8	4,237
Thurrock	37.6	5,690
Barnsley	37.4	5,560
Hillingdon	36.8	9,755
Basildon	36.8	5,752
Dartford	36.6	2,888
Havering	36.5	8,012
Luton	36.5	5,089
Sandwell	36.5	9,165
St. Helens	36.4	5,505
Bolsover	36.4	73
Medway	36.3	9,291
Mansfield	36.2	2,273
Crawley	36.1	2,499
Gravesham	36.1	2,506
Rotherham	36.1	5,677
Wakefield	36.1	6,635
Redcar and Cleveland	35.9	3,176

Data source: GPPS respondents in full-time employment, 2012-2017

Moss C, *et al. BMJ Open* 2022; 12:e062942. doi: 10.1136/bmjopen-2022-062942

Supplementary Table 4: Descriptive statistics: Percentage of respondents who gave each response for each EQ5D domain

	**Mobility**		**Self-care**		**Usual activities**		**Pain/discomfort**		**Anxiety/ depression**	
	**Female**	**Male**	**Female**	**Male**	**Female**	**Male**	**Female**	**Male**	**Female**	**Male**
1. No problems	91.16	90.47	98.44	98.35	87.74	88.02	63.34	63.81	71.17	77.21
2. Slight problems	6.49	6.98	1.09	1.15	9.27	8.99	26.35	27.15	20.00	15.98
3. Moderate problems	1.80	1.95	0.35	0.34	2.37	2.35	8.40	7.51	7.31	5.63
4. Severe problems	0.43	0.44	0.06	0.06	0.45	0.46	1.69	1.32	1.19	0.89
5. Unable to/extreme problems	0.12	0.16	0.06	0.09	0.18	0.19	0.23	0.20	0.33	0.27

Data source: GPPS respondents in full-time employment, 2012-2017

Moss C, *et al. BMJ Open* 2022; 12:e062942. doi: 10.1136/bmjopen-2022-062942

Supplementary Table 5: Descriptive statistics: Prevalence of long-term conditions (%)

	**Female**	**Male**
Alzheimers disease or dementia	0.02	0.04
Angina or long-term heart problem	0.57	1.64
Arthritis or long-term joint problem	4.37	3.74
Asthma or long-term chest problem	8.85	7.83
Blindness or severe visual impairment	0.12	0.21
Cancer in the last 5 years	1.37	0.99
Deafness or severe hearing impairment	0.72	1.11
Diabetes	2.21	4.22
Epilepsy	0.54	0.59
High blood pressure	6.14	9.67
Kidney or liver disease	0.57	0.78
Learning difficulty	0.09	0.17
Long-term back problem	5.12	5.74
Long-term mental health problem	3.29	1.97
Long-term neurological problem	0.89	0.67
Another long-term condition	10.14	8.01
Prefer not to say	1.77	1.91
**N**	570,626	662,258

Data source: GPPS respondents in full-time employment, 2012-2017

Supplementary Table 6: The effect of covariates on reporting an inflexible job and the effect of an inflexible job on the EQ-5D utility score; full sample of male and female, with interaction effects

	Inflexible job<br>(1)	EQ-5D<br>(2)
Inflexible job		-0.009
		[-0.012; -0.006]
Female	0.060	-0.010
	[0.058; 0.062]	[-0.011; -0.010]
Female * inflexible job		-0.002
		[-0.004; -0.001]
Parent	-0.001	0.013
	[-0.003; 0.002]	[0.012; 0.014]
Aged 25-34 years [a]	-0.063	-0.009
	[-0.068; -0.057]	[-0.011; -0.007]
Aged 35-44 years [a]	-0.093	-0.028
	[-0.098; -0.088]	[-0.029; -0.026]
Aged 45-54 years [a]	-0.104	-0.045
	[-0.109; -0.099]	[-0.046; -0.043]
Aged 55-64 years [a]	-0.133	-0.058
	[-0.138; -0.128]	[-0.060; -0.057]
Aged 25-34 years * inflexible job		-0.000
		[-0.003; 0.003]
Aged 35-44 years * inflexible job		-0.003
		[-0.006; -0.000]
Aged 45-54 years * inflexible job		-0.007
		[-0.010; -0.004]
Aged 55-64 years * inflexible job		-0.010
		[-0.013; -0.007]
Ethnicity: mixed [b]	0.001	-0.007
	[-0.009; 0.012]	[-0.010; -0.004]
Ethnicity: Asian [b]	0.018	0.007
	[0.014; 0.023]	[0.005; 0.008]
Ethnicity: black [b]	-0.020	0.010
	[-0.026; -0.013]	[0.008; 0.012]
Ethnicity: other [b]	0.011	-0.006
	[0.003; 0.019]	[-0.009; -0.003]
Never smoked	-0.011	0.026
	[-0.013; -0.008]	[0.025; 0.026]
Deprivation decile 2 [c]	0.006	-0.003
(least deprived)	[0.001; 0.010]	[-0.005; -0.002]
Deprivation decile 3 [c]	0.008	-0.006
	[0.003; 0.012]	[-0.008; -0.005]
Deprivation decile 4 [c]	0.018	-0.008
	[0.013; 0.022]	[-0.009; -0.006]
Deprivation decile 5 [c]	0.022	-0.009

	[0.017; 0.027]	[-0.011; -0.008]
Deprivation decile 6 [c]	0.028	-0.014
	[0.023; 0.033]	[-0.015; -0.012]
Deprivation decile 7 [c]	0.036	-0.017
	[0.031; 0.041]	[-0.019; -0.016]
Deprivation decile 8 [c]	0.043	-0.020
	[0.038; 0.048]	[-0.022; -0.018]
Deprivation decile 9 [c]	0.059	-0.022
	[0.053; 0.064]	[-0.024; -0.021]
Deprivation decile 10 [c]	0.072	-0.028
(most deprived)	[0.066; 0.077]	[-0.030; -0.026]
Deprivation decile 2 * inflexible job		0.001
(least deprived)		[-0.002; 0.004]
Deprivation decile 3 * inflexible job		0.003
		[-0.000; 0.006]
Deprivation decile 4 * inflexible job		0.000
		[-0.003; 0.003]
Deprivation decile 5 * inflexible job		-0.003
		[-0.006; -0.000]
Deprivation decile 6 * inflexible job		-0.002
		[-0.005; 0.001]
Deprivation decile 7 * inflexible job		-0.005
		[-0.008; -0.002]
Deprivation decile 8 * inflexible job		-0.004
		[-0.007; -0.001]
Deprivation decile 9 * inflexible job		-0.005
		[-0.008; -0.002]
Deprivation decile 10 * inflexible job		-0.006
(most deprived)		[-0.010; -0.003]
N	1,232,884	1,232,884

Data source: GPPS pooled data for 2012 to 2017.
95% confidence intervals in parentheses.
Models estimated using OLS.
[a] The reference category for age group is 16-24 years;
[b] The reference category for ethnic group is white;
[c] The reference category for deprivation is decile 1;
Models additionally include fixed effects for survey year and Local Authority District of residence.

Moss C, *et al. BMJ Open* 2022; 12:e062942. doi: 10.1136/bmjopen-2022-062942

Supplementary Table 7: The effect of covariates on reporting an inflexible job and the effect of an inflexible job on the EQ-5D utility score; full-time and part-time workers

	Inflexible job		EQ-5D	
	Females (1)	Males (2)	Females (3)	Males (4)
Inflexible job			-0.018	-0.016
			[-0.018; -0.017]	[-0.017; -0.015]
Part-time	0.027	-0.017	-0.016	-0.039
	[0.025; 0.030]	[-0.021; -0.012]	[-0.017; -0.015]	[-0.040; -0.037]
Parent	-0.005	0.002	0.025	0.012
	[-0.008; -0.002]	[-0.002; 0.005]	[0.024; 0.025]	[0.011; 0.013]
Aged 25-34 years [a]	-0.054	-0.060	-0.000	-0.023
	[-0.060; -0.048]	[-0.067; -0.052]	[-0.002; 0.001]	[-0.025; -0.021]
Aged 35-44 years [a]	-0.076	-0.088	-0.020	-0.042
	[-0.082; -0.070]	[-0.095; -0.080]	[-0.022; -0.018]	[-0.044; -0.040]
Aged 45-54 years [a]	-0.086	-0.100	-0.039	-0.061
	[-0.092; -0.080]	[-0.107; -0.093]	[-0.040; -0.037]	[-0.062; -0.059]
Aged 55-64 years [a]	-0.117	-0.131	-0.047	-0.073
	[-0.123; -0.111]	[-0.138; -0.124]	[-0.049; -0.045]	[-0.074; -0.071]
Ethnicity: mixed [b]	0.001	0.006	-0.014	-0.006
	[-0.010; 0.013]	[-0.008; 0.020]	[-0.017; -0.010]	[-0.011; -0.002]
Ethnicity: Asian [b]	-0.002	0.017	0.001	0.008
	[-0.007; 0.004]	[0.011; 0.022]	[-0.001; 0.003]	[0.007; 0.010]
Ethnicity: black [b]	-0.037	-0.010	-0.002	0.020
	[-0.045; -0.030]	[-0.019; -0.002]	[-0.004; 0.000]	[0.017; 0.022]
Ethnicity: other [b]	-0.016	0.007	-0.013	-0.004
	[-0.025; -0.006]	[-0.002; 0.015]	[-0.017; -0.010]	[-0.007; -0.000]
Never smoked	0.009	-0.022	0.028	0.026
	[0.007; 0.012]	[-0.025; -0.019]	[0.027; 0.028]	[0.025; 0.027]
Deprivation decile 1 [c]	-0.005	-0.029	0.012	0.012
(least deprived)	[-0.011; 0.001]	[-0.035; -0.023]	[0.010; 0.014]	[0.010; 0.014]
Deprivation decile 2 [c]	-0.009	-0.019	0.007	0.008
	[-0.014; -0.003]	[-0.025; -0.013]	[0.006; 0.009]	[0.006; 0.010]
Deprivation decile 3 [c]	-0.007	-0.018	0.005	0.006
	[-0.012; -0.001]	[-0.024; -0.012]	[0.004; 0.007]	[0.004; 0.008]
Deprivation decile 4 [c]	-0.005	-0.005	0.002	0.003
	[-0.010; 0.000]	[-0.012; 0.001]	[0.000; 0.004]	[0.001; 0.005]
Deprivation decile 6 [c]	0.007	0.007	-0.005	-0.003
	[0.002; 0.013]	[0.001; 0.013]	[-0.007; -0.004]	[-0.005; -0.002]
Deprivation decile 7 [c]	0.009	0.016	-0.010	-0.007
	[0.004; 0.015]	[0.010; 0.022]	[-0.012; -0.008]	[-0.009; -0.006]
Deprivation decile 8 [c]	0.014	0.024	-0.015	-0.010
	[0.009; 0.020]	[0.017; 0.030]	[-0.016; -0.013]	[-0.012; -0.008]
Deprivation decile 9 [c]	0.026	0.039	-0.018	-0.013

	[0.020; 0.032]	[0.032; 0.045]	[-0.020; -0.017]	[-0.015; -0.010]
Deprivation decile 10 [c]	0.037	0.049	-0.025	-0.017
(most deprived)	[0.030; 0.043]	[0.042; 0.056]	[-0.027; -0.024]	[-0.020; -0.015]
N	912,598	742,534	912,598	742,534

Data source: GPPS pooled data for 2012 to 2017.
95% confidence intervals in parentheses.
Models estimated using OLS.
[a] The reference category for age group is 16-24 years;
[b] The reference category for ethnic group is white;
[c] The reference category for deprivation is decile 5;
Models additionally include fixed effects for survey year and Local Authority District of residence.

Supplementary Table 8: The effect of an inflexible job on healthcare utilisation

	**Months since last saw GP**		**Months since last saw nurse**	
	Females	Males	Females	Males
	(1)	(2)	(3)	(4)
Inflexible job	0.234	0.199	0.569	0.653
	[0.201; 0.268]	[0.152; 0.247]	[0.519; 0.618]	[0.586; 0.720]
Parent	-1.341	-0.926	-1.397	-0.307
	[-1.377; -1.305]	[-0.975; -0.877]	[-1.450; -1.343]	[-0.376; -0.239]
Aged 25-34 years [a]	0.202	-0.053	-1.097	-0.251
	[0.132; 0.272]	[-0.175; 0.069]	[-1.208; -0.986]	[-0.423; -0.080]
Aged 35-44 years [a]	0.830	-0.357	-0.206	-1.130
	[0.759; 0.901]	[-0.475; -0.239]	[-0.317; -0.094]	[-1.297; -0.963]
Aged 45-54 years [a]	1.055	-0.571	0.497	-2.088
	[0.988; 1.122]	[-0.684; -0.457]	[0.391; 0.604]	[-2.248; -1.928]
Aged 55-64 years [a]	1.233	-1.031	0.933	-2.990
	[1.161; 1.305]	[-1.145; -0.918]	[0.820; 1.045]	[-3.151; -2.829]
Ethnicity: mixed [b]	-0.135	-0.325	-0.205	-0.521
	[-0.273; 0.002]	[-0.550; -0.100]	[-0.418; 0.008]	[-0.853; -0.189]
Ethnicity: Asian [b]	-0.034	-1.151	0.420	-1.523
	[-0.108; 0.040]	[-1.237; -1.065]	[0.304; 0.537]	[-1.649; -1.397]
Ethnicity: black [b]	-0.492	-1.133	-0.436	-2.228
	[-0.573; -0.410]	[-1.255; -1.010]	[-0.572; -0.300]	[-2.412; -2.044]
Ethnicity: other [b]	-0.035	-0.888	-0.167	-1.947
	[-0.173; 0.103]	[-1.035; -0.742]	[-0.366; 0.032]	[-2.157; -1.737]
Never smoked	0.402	0.359	0.329	0.305
	[0.370; 0.435]	[0.317; 0.402]	[0.281; 0.378]	[0.245; 0.364]
Deprivation decile 1 [c]	-0.014	0.030	0.017	0.154
(least deprived)	[-0.087; 0.058]	[-0.066; 0.126]	[-0.091; 0.124]	[0.021; 0.287]
Deprivation decile 2 [c]	-0.038	0.001	0.024	0.086
	[-0.107; 0.031]	[-0.092; 0.095]	[-0.080; 0.128]	[-0.044; 0.216]
Deprivation decile 3 [c]	0.022	-0.034	0.082	0.093
	[-0.048; 0.092]	[-0.128; 0.060]	[-0.021; 0.185]	[-0.037; 0.222]
Deprivation decile 4 [c]	-0.044	-0.023	-0.025	0.034
	[-0.113; 0.025]	[-0.117; 0.070]	[-0.127; 0.077]	[-0.096; 0.164]
Deprivation decile 6 [c]	0.029	-0.028	0.067	0.001
	[-0.041; 0.098]	[-0.124; 0.067]	[-0.037; 0.170]	[-0.132; 0.133]
Deprivation decile 7 [c]	-0.093	-0.013	0.005	0.030
	[-0.163; -0.023]	[-0.109; 0.084]	[-0.099; 0.110]	[-0.105; 0.165]
Deprivation decile 8 [c]	-0.040	0.033	0.033	-0.045
	[-0.112; 0.031]	[-0.065; 0.131]	[-0.073; 0.140]	[-0.181; 0.092]
Deprivation decile 9 [c]	-0.003	-0.031	-0.062	-0.084
	[-0.077; 0.070]	[-0.132; 0.070]	[-0.171; 0.047]	[-0.224; 0.057]
Deprivation decile 10 [c]	-0.062	-0.070	0.028	-0.124
(most deprived)	[-0.140; 0.015]	[-0.174; 0.034]	[-0.087; 0.143]	[-0.269; 0.021]

Appointment availability	-2.756	-3.262	0.338	1.374
	[-3.077; -2.436]	[-3.643; -2.880]	[-0.099; 0.774]	[0.850; 1.899]
Alzheimers	1.320	2.788	0.650	2.558
	[0.034; 2.607]	[1.134; 4.442]	[-1.147; 2.447]	[0.246; 4.871]
Angina	-0.944	-2.177	-1.391	-3.777
	[-1.109; -0.779]	[-2.282; -2.073]	[-1.632; -1.149]	[-3.935; -3.619]
Arthritis	-1.247	-1.707	-0.925	-1.254
	[-1.302; -1.192]	[-1.783; -1.631]	[-1.018; -0.831]	[-1.378; -1.131]
Asthma	-1.098	-1.955	-2.312	-4.488
	[-1.144; -1.053]	[-2.022; -1.888]	[-2.381; -2.243]	[-4.584; -4.392]
Blindness	-0.004	0.587	1.281	1.035
	[-0.387; 0.380]	[0.104; 1.070]	[0.494; 2.068]	[0.351; 1.719]
Cancer in past 5 years	-1.361	-2.347	-1.050	-2.956
	[-1.460; -1.263]	[-2.487; -2.207]	[-1.212; -0.888]	[-3.189; -2.723]
Deafness	-0.380	-0.537	-0.290	-0.250
	[-0.538; -0.222]	[-0.693; -0.380]	[-0.540; -0.039]	[-0.488; -0.011]
Diabetes	-1.387	-2.579	-3.767	-6.546
	[-1.460; -1.315]	[-2.644; -2.515]	[-3.863; -3.670]	[-6.626; -6.465]
Epilepsy	-1.253	-2.297	-0.510	-1.799
	[-1.404; -1.102]	[-2.523; -2.072]	[-0.828; -0.193]	[-2.211; -1.387]
High blood pressure	-1.295	-2.339	-2.486	-4.421
	[-1.344; -1.247]	[-2.387; -2.292]	[-2.558; -2.415]	[-4.489; -4.352]
Kidney/liver disease	-0.819	-1.435	-1.200	-2.038
	[-0.984; -0.655]	[-1.612; -1.258]	[-1.446; -0.955]	[-2.331; -1.744]
Learning difficulties	0.677	0.352	0.648	-0.243
	[-0.038; 1.392]	[-0.261; 0.964]	[-0.421; 1.717]	[-1.086; 0.600]
Back problem	-0.896	-1.368	-0.134	-0.511
	[-0.953; -0.839]	[-1.439; -1.297]	[-0.233; -0.034]	[-0.627; -0.395]
Mental health	-1.864	-3.461	-0.231	-1.379
	[-1.922; -1.806]	[-3.566; -3.355]	[-0.362; -0.101]	[-1.599; -1.160]
Neurological	-1.405	-2.161	-0.832	-1.745
	[-1.515; -1.295]	[-2.350; -1.972]	[-1.047; -0.617]	[-2.079; -1.411]
Another condition	-1.574	-2.569	-1.445	-2.638
	[-1.613; -1.534]	[-2.627; -2.512]	[-1.514; -1.376]	[-2.735; -2.540]
Prefer not to say	-1.622	-1.927	-0.607	-1.734
	[-1.724; -1.521]	[-2.081; -1.773]	[-0.796; -0.419]	[-1.968; -1.499]
N	570,626	662,258	570,626	662,258

Data source: GPPS pooled data for 2012 to 2017.
95% confidence intervals in parentheses.
Models estimated using interval regression.
[a] The reference category for age group is 16-24 years;
[b] The reference category for ethnic group is white;
[c] The reference category for deprivation is decile 5;
Models additionally include fixed effects for survey year and Local Authority District of residence.

Moss C, *et al. BMJ Open* 2022; 12:e062942. doi: 10.1136/bmjopen-2022-062942

Supplementary Table 9: Sensitivity analyses: altering the intervals in health care use regressions

<table>
<tr><th>Survey response category</th><th colspan="3">Intervals (months since last seen GP/nurse)</th><th>Extensive margin: never seen GP/nurse before = 1</th></tr>
<tr><th></th><th>Main analysis</th><th>Sensitivity 1</th><th>Sensitivity 2</th><th>Sensitivity 3</th></tr>
<tr><td>In the past 3 months</td><td>0 to 3</td><td>0 to 3</td><td>0 to 3</td><td rowspan="4">Have seen before</td></tr>
<tr><td>Between 3 and 6 months ago</td><td>3 to 6</td><td>3 to 6</td><td>3 to 6</td></tr>
<tr><td>Between 6 and 12 months ago</td><td>6 to 12</td><td>6 to 12</td><td>6 to 12</td></tr>
<tr><td>More than 12 months ago</td><td>12 to 24</td><td>12 to 60</td><td rowspan="2">12+</td></tr>
<tr><td>I have never seen a GP/nurse from my GP surgery</td><td>24+</td><td>60+</td><td>Never seen before</td></tr>
</table>

General Practice Patient Survey, Questions 1 and 2

Supplementary Table 10: Results of sensitivity analyses altering the intervals in health care use regressions: coefficient on inflexible job

	Months since last saw GP		Months since last saw nurse	
	Female (1)	Males (2)	Females (3)	Males (4)
**Main analysis**				
Inflexible job	0.234	0.199	0.569	0.653
	[0.201; 0.268]	[0.152; 0.247]	[0.519; 0.618]	[0.586; 0.720]
**Sensitivity 1**				
Inflexible job	0.288	0.316	1.056	1.783
	[0.232; 0.344]	[0.222; 0.409]	[0.947; 1.165]	[1.600; 1.970]
**Sensitivity 2**				
Inflexible job	0.221	0.180	0.486	0.459
	[0.192; 0.251]	[0.138; 0.221]	[0.445; 0.527]	[0.402; 0.516]
**Sensitivity 3**				
Inflexible job	0.001	0.001	0.006	0.021
	[0.0001; 0.002]	[0.0002; 0.003]	[0.005; 0.008]	[0.018; 0.023]
N	570,626	662,258	570,626	662,258

Data source: GPPS pooled data for 2012 to 2017.
95% confidence intervals in parentheses.
All models additionally include indicators for: parental status, ten-year age group, ethnicity, whether the respondent has ever smoked, index of multiple deprivation decile, 16 long-term conditions, practice level appointment availability, and fixed effects for survey year and Local Authority District of residence.
Main analysis, Sensitivity 1 and Sensitivity 2 estimated using interval regression.
Sensitivity 3 models (1), (3) and (4) estimated using logistic regression, results show marginal effects.
Sensitivity 3 model (2) estimated using OLS due to non-convergence of logit model.

Supplementary Table 11: The effect of an inflexible job on healthcare utilisation: out-of hours

	Females (1)	Males (2)
Inflexible job	0.197	0.234
	[0.157,0.238]	[0.184,0.284]
Parent	0.313	0.306
	[0.266,0.361]	[0.253,0.359]
Aged 25-34 years [a]	-0.352	-0.215
	[-0.422,-0.282]	[-0.325,-0.105]
Aged 35-44 years [a]	-0.768	-0.497
	[-0.843,-0.694]	[-0.606,-0.387]
Aged 45-54 years [a]	-0.977	-0.724
	[-1.048,-0.906]	[-0.831,-0.617]
Aged 55-64 years [a]	-1.074	-0.83
	[-1.157,-0.990]	[-0.942,-0.717]
Ethnicity: mixed [b]	0.081	0.024
	[-0.095,0.258]	[-0.213,0.261]
Ethnicity: Asian [b]	0.241	0.347
	[0.153,0.328]	[0.262,0.432]
Ethnicity: black [b]	0.074	-0.033
	[-0.047,0.194]	[-0.194,0.129]
Ethnicity: other [b]	0.395	0.66
	[0.265,0.525]	[0.547,0.773]
Never smoked	-0.126	-0.068
	[-0.167,-0.084]	[-0.116,-0.020]
Deprivation decile 1 [c]	-0.009	-0.089
(least deprived)	[-0.107,0.090]	[-0.205,0.026]
Deprivation decile 2 [c]	0.046	-0.142
	[-0.049,0.141]	[-0.257,-0.028]
Deprivation decile 3 [c]	-0.008	0.016
	[-0.101,0.085]	[-0.095,0.127]
Deprivation decile 4 [c]	0.067	0.028
	[-0.025,0.159]	[-0.081,0.137]
Deprivation decile 6 [c]	0.097	0.035
	[0.006,0.189]	[-0.077,0.147]
Deprivation decile 7 [c]	0.074	0.081
	[-0.017,0.166]	[-0.029,0.191]
Deprivation decile 8 [c]	0.036	0.123
	[-0.058,0.129]	[0.008,0.237]
Deprivation decile 9 [c]	0.108	0.189
	[0.013,0.204]	[0.075,0.303]
Deprivation decile 10 [c]	0.148	0.272
(most deprived)	[0.050,0.245]	[0.158,0.385]
Appointment availability	-0.898	-1.088
	[-1.241,-0.556]	[-1.479,-0.697]

Alzheimers	1.35	2.12
	[0.410,2.289]	[1.457,2.783]
Angina	0.497	0.584
	[0.306,0.689]	[0.447,0.722]
Arthritis	0.213	0.198
	[0.125,0.301]	[0.095,0.301]
Asthma	0.505	0.4
	[0.445,0.565]	[0.323,0.476]
Blindness	-0.262	0.035
	[-0.883,0.359]	[-0.520,0.590]
Cancer in past 5 years	0.481	0.441
	[0.338,0.623]	[0.258,0.624]
Deafness	0.27	0.036
	[0.059,0.480]	[-0.162,0.234]
Diabetes	0.446	0.387
	[0.330,0.561]	[0.295,0.478]
Epilepsy	0.233	0.272
	[-0.003,0.469]	[0.002,0.542]
High blood pressure	0.107	0.152
	[0.026,0.187]	[0.079,0.225]
Kidney/liver disease	0.471	0.629
	[0.270,0.672]	[0.437,0.822]
Learning difficulties	0.116	0.34
	[-0.284,0.516]	[-0.004,0.684]
Back problem	0.25	0.472
	[0.171,0.330]	[0.388,0.557]
Mental health	0.405	0.569
	[0.305,0.504]	[0.418,0.720]
Neurological	0.657	0.424
	[0.488,0.826]	[0.202,0.647]
Another condition	0.511	0.522
	[0.455,0.568]	[0.451,0.593]
Prefer not to say	0.445	0.507
	[0.309,0.582]	[0.357,0.657]
N	293,266	336,890

Data source: GPPS pooled data for 2012 to 2014.
95% confidence intervals in parentheses.
Models estimated using logistic regression.
[a] The reference category for age group is 16-24 years;
[b] The reference category for ethnic group is white;
[c] The reference category for deprivation is decile 5;
Models additionally include fixed effects for survey year and Local Authority District of residence.

Moss C, *et al. BMJ Open* 2022; 12:e062942. doi: 10.1136/bmjopen-2022-062942

Supplementary Table 12: Two-stage least squares regression results: the mediating effect of nurse visits in the effect of an inflexible job on health

	**First stage: Time since last saw nurse**		**Second stage: EQ-5D utility score**	
	Females	Males	Females	Males
	(1)	(2)	(3)	(4)
Time since last saw nurse			-0.175	-0.207
			[-0.195,-0.155]	[-0.238,-0.177]
Inflexible job	0.095	0.076		
	[0.086,0.103]	[0.066,0.085]		
Parent	-0.232	-0.019	-0.023	0.007
	[-0.242,-0.222]	[-0.029,-0.010]	[-0.028,-0.018]	[0.004,0.009]
Aged 25-34 years [a]	-0.174	-0.053	-0.029	-0.030
	[-0.193,-0.155]	[-0.075,-0.030]	[-0.034,-0.024]	[-0.036,-0.024]
Aged 35-44 years [a]	-0.022	-0.223	-0.023	-0.084
	[-0.041,-0.003]	[-0.245,-0.201]	[-0.027,-0.019]	[-0.093,-0.075]
Aged 45-54 years [a]	0.066	-0.454	-0.026	-0.150
	[0.048,0.084]	[-0.475,-0.433]	[-0.030,-0.023]	[-0.165,-0.135]
Aged 55-64 years [a]	0.056	-0.767	-0.040	-0.230
	[0.037,0.075]	[-0.789,-0.746]	[-0.044,-0.036]	[-0.254,-0.206]
Ethnicity: mixed [b]	-0.055	-0.098	-0.019	-0.025
	[-0.092,-0.017]	[-0.145,-0.051]	[-0.028,-0.011]	[-0.036,-0.013]
Ethnicity: Asian [b]	0.049	-0.278	0.013	-0.050
	[0.030,0.069]	[-0.296,-0.260]	[0.009,0.018]	[-0.059,-0.041]
Ethnicity: black [b]	-0.111	-0.336	-0.019	-0.052
	[-0.135,-0.088]	[-0.363,-0.308]	[-0.024,-0.013]	[-0.064,-0.040]
Ethnicity: other [b]	-0.077	-0.334	-0.023	-0.074
	[-0.111,-0.043]	[-0.365,-0.304]	[-0.031,-0.016]	[-0.086,-0.061]
Never smoked	0.069	0.072	0.038	0.040
	[0.060,0.077]	[0.064,0.081]	[0.036,0.041]	[0.037,0.043]
Deprivation decile 1 [c]	0.023	0.053	0.013	0.022
(least deprived)	[0.004,0.042]	[0.034,0.072]	[0.009,0.017]	[0.017,0.027]
Deprivation decile 2 [c]	0.015	0.035	0.008	0.015
	[-0.004,0.033]	[0.016,0.054]	[0.004,0.012]	[0.011,0.020]
Deprivation decile 3 [c]	0.021	0.029	0.008	0.012
	[0.003,0.039]	[0.010,0.047]	[0.004,0.012]	[0.007,0.016]
Deprivation decile 4 [c]	-0.003	0.012	0.001	0.006
	[-0.021,0.016]	[-0.006,0.031]	[-0.003,0.005]	[0.001,0.010]
Deprivation decile 6 [c]	0.003	-0.003	-0.004	-0.004
	[-0.016,0.021]	[-0.022,0.016]	[-0.008,-0.000]	[-0.008,0.001]
Deprivation decile 7 [c]	-0.009	-0.014	-0.011	-0.011
	[-0.028,0.009]	[-0.034,0.005]	[-0.015,-0.007]	[-0.015,-0.006]
Deprivation decile 8 [c]	-0.010	-0.032	-0.014	-0.016
	[-0.029,0.009]	[-0.052,-0.012]	[-0.018,-0.010]	[-0.021,-0.012]
Deprivation decile 9 [c]	-0.032	-0.043	-0.021	-0.022

Moss C, *et al. BMJ Open* 2022; 12:e062942. doi: 10.1136/bmjopen-2022-062942

	[-0.051,-0.012]	[-0.063,-0.023]	[-0.026,-0.017]	[-0.027,-0.017]
Deprivation decile 10 [c]	-0.025	-0.073	-0.027	-0.033
(most deprived)	[-0.046,-0.005]	[-0.094,-0.052]	[-0.032,-0.023]	[-0.038,-0.027]
N	570,626	662,258	570,626	662,258

Data source: GPPS pooled data for 2012 to 2017.
95% confidence intervals in parentheses.
[a] The reference category for age group is 16-24 years.
[b] The reference category for ethnic group is white.
[c] The reference category for deprivation is decile 5.
Models additionally include fixed effects for survey year and Local Authority District of residence.

Supplementary Figure 1: Flowchart of observations dropped at each stage of data preparation

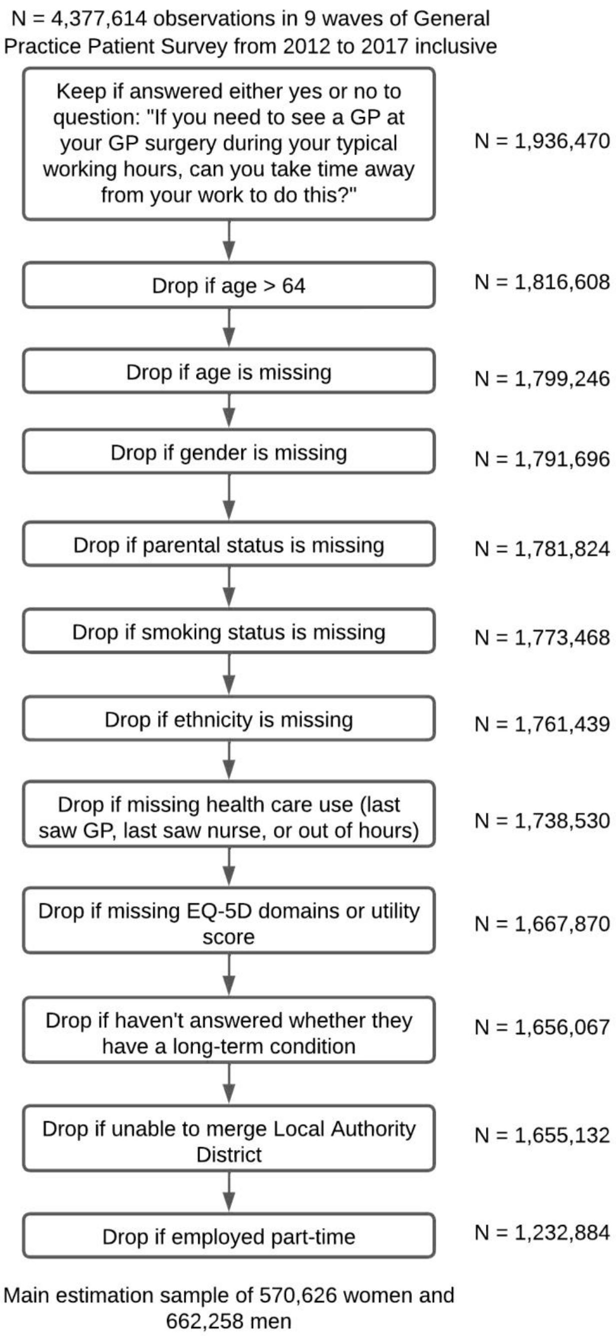

Moss C, *et al. BMJ Open* 2022; 12:e062942. doi: 10.1136/bmjopen-2022-062942

Supplementary Figure 2: Geographic distribution of inflexible jobs and health-related quality of life in England

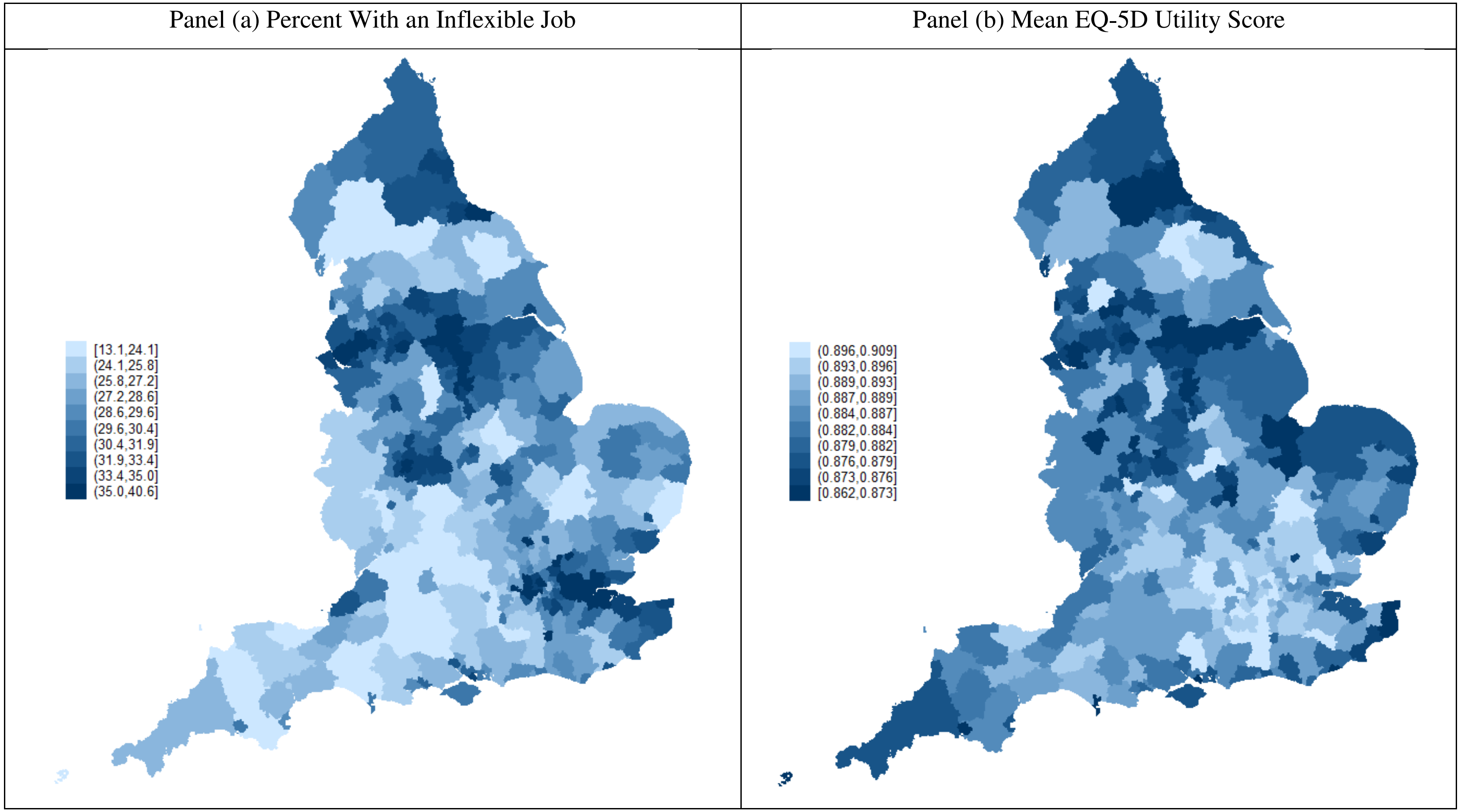

Data source: 1,346,001 General Practice Patient Survey respondents in full-time employment who have complete data for questions relating to job flexibility and EQ-5D utility score; pooled data from 2012-2017; respondents who report inability to visit their GP during working hours are classified as having an inflexible job; averages for 326 local authority districts; maps created using spmap (Pisati, 2018); deciles generated within spmap
