# Supplementary material for: Associations between inflexible job conditions, health and healthcare utilisation in England: retrospective cross-sectional study
Source: BMJ Open. 2022 Dec 5;12(12):e062942. doi: 10.1136/bmjopen-2022-062942 (PMC9723827; doi:10.1136/bmjopen-2022-062942)
Supplement: Author's manuscript [file bmjopen-2022-062942.draft_revisions.pdf]

BMJ Open is committed to open peer review. As part of this commitment we make the peer review history of every article we publish publicly available.

When an article is published we post the peer reviewers' comments and the authors' responses online. We also post the versions of the paper that were used during peer review. These are the versions that the peer review comments apply to.

The versions of the paper that follow are the versions that were submitted during the peer review process. They are not the versions of record or the final published versions. They should not be cited or distributed as the published version of this manuscript.

BMJ Open is an open access journal and the full, final, typeset and author-corrected version of record of the manuscript is available on our site with no access controls, subscription charges or pay-per-view fees (http://bmjopen.bmj.com).

If you have any questions on BMJ Open's open peer review process please email info.bmjopen@bmj.com

Health, health care utilisation and inflexible job conditions: retrospective cross-sectional study

Journal:	*BMJ Open*
Manuscript ID	bmjopen-2022-062942
Article Type:	Original research
Date Submitted by the Author:	16-Mar-2022

Complete List of Authors:	Moss, Charlie; The University of Manchester, Health Organisation, Policy and Economics (HOPE), Centre for Primary Care and Health Services Research, School of Health Sciences<br>Munford, Luke; The University of Manchester, Health Organisation, Policy and Economics (HOPE), Centre for Primary Care and Health Services Research, School of Health Sciences<br>Sutton, Matt; The University of Manchester, Health Organisation, Policy and Economics (HOPE), Centre for Primary Care and Health Services Research, School of Health Sciences; The University of Melbourne, Melbourne Institute: Applied Economic and Social Research

Keywords:	PUBLIC HEALTH, OCCUPATIONAL & INDUSTRIAL MEDICINE, PRIMARY CARE, SOCIAL MEDICINE

SCHOLARONE™
Manuscripts

I, the Submitting Author has the right to grant and does grant on behalf of all authors of the Work (as defined in the below author licence), an exclusive licence and/or a non-exclusive licence for contributions from authors who are: i) UK Crown employees; ii) where BMJ has agreed a CC-BY licence shall apply, and/or iii) in accordance with the terms applicable for US Federal Government officers or employees acting as part of their official duties; on a worldwide, perpetual, irrevocable, royalty-free basis to BMJ Publishing Group Ltd ("BMJ") its licensees and where the relevant Journal is co-owned by BMJ to the co-owners of the Journal, to publish the Work in this journal and any other BMJ products and to exploit all rights, as set out in our licence.

The Submitting Author accepts and understands that any supply made under these terms is made by BMJ to the Submitting Author unless you are acting as an employee on behalf of your employer or a postgraduate student of an affiliated institution which is paying any applicable article publishing charge ("APC") for Open Access articles. Where the Submitting Author wishes to make the Work available on an Open Access basis (and intends to pay the relevant APC), the terms of reuse of such Open Access shall be governed by a Creative Commons licence – details of these licences and which Creative Commons licence will apply to this Work are set out in our licence referred to above.

Other than as permitted in any relevant BMJ Author's Self Archiving Policies, I confirm this Work has not been accepted for publication elsewhere, is not being considered for publication elsewhere and does not duplicate material already published. I confirm all authors consent to publication of this Work and authorise the granting of this licence.

For peer review only

Health, health care utilisation and inflexible job conditions: retrospective cross-sectional study

Charlie Moss,[1] Luke Munford,[1] Matt Sutton,[1,2]

[1] Health Organisation, Policy and Economics (HOPE), Centre for Primary Care and Health Services Research, School of Health Sciences, The University of Manchester, Manchester, UK

[2] Melbourne Institute: Applied Economic and Social Research, The University of Melbourne, Melbourne, Australia

Corresponding author: Charlie Moss, 6.27 Williamson Building, The University of Manchester, Oxford Road, Manchester, M13 9PL, UK; charlie.moss@manchester.ac.uk

For peer review only

For peer review only - http://bmjopen.bmj.com/site/about/guidelines.xhtml

ABSTRACT

Objectives: To estimate the strength of association between having an inflexible job and health-related quality of life and health care utilisation; and to explore heterogeneity in the effects by gender, age and area-level deprivation.

Design: Retrospective cross-sectional study.

Setting: Seven waves of the English General Practice Patient Survey between 2012 and 2017.

Participants: 1,173,225 people aged 16 to 64 and in full-time employment.

Methods: We measured job inflexibility by inability to take time away from work during usual working hours to seek medical care. We used linear regression to estimate the strength of association between outcomes and having an inflexible job, adjusting for person and area-level characteristics.

Primary and secondary outcome measures: Health-related quality of life (EQ-5D-5L); number of months since the respondent last saw a GP or nurse; use of out-of-hours general practice in the past six months.

Results: One-third of respondents reported job inflexibility. The probability of job inflexibility was higher at younger ages and in more deprived areas. Job inflexibility was associated with lower EQ-5D-5L utility scores of 0.017 (95% confidence interval (CI) [0.016; 0.018]) for women and 0.016 (95% CI [0.015; 0.017]) for men. Men were more affected in physical health domains and women in the mental health domain. The reduction in health-related quality of life associated with having an inflexible job was greater for employees who were older or lived in more deprived areas. Having an inflexible job was also associated with a longer time since the last visit to their GP of 0.178 (95% CI [0.145; 0.212]) months for women and 0.146 (95% CI [0.010; 0.192]) months for men.

Conclusions: Inequalities in the prevalence of inflexible jobs contribute to inequalities in health. Policymakers and employers should ensure that all employees have sufficient job flexibility to protect their health.

Keywords: work; health; health care; job flexibility

For peer review only - http://bmjopen.bmj.com/site/about/guidelines.xhtml

Strengths and limitations of this study

- Large sample of 1,173,225 survey respondents.
- We were able to use the EQ-5D-5L instrument which is a validated measure of health-related quality of life.
- The GPPS is a repeated cross-sectional survey. This makes it difficult to establish causality.
- The measure of job flexibility may not fully capture other time constraints that are unrelated to health care utilisation during working hours.

For peer review only

For peer review only - http://bmjopen.bmj.com/site/about/guidelines.xhtml

INTRODUCTION

Employee-oriented work flexibility refers to workers' discretion over some aspect of their job, such as location or hours of work. Examples include "flexitime", involving discretion in start and finish times, and the option to work from home. This should improve working and social life for the employee by increasing time sovereignty.[1] Enabling individuals to adjust the working day to do other important tasks, such as health promoting behaviours and consuming medical care, is important for workplace health and productivity.

The literature suggests that work flexibility may be positively associated with health and negatively associated with burnout, perceived stress, sickness absence,[1–8] but it suffers from several limitations. It is based predominantly on work-focused surveys that include only crude measures of health and no information on healthcare utilisation. There is limited evidence of variation between population subgroups, even though we may expect some groups of employees to gain more from work flexibility (older employees and employees with other responsibilities).[9]

We consider full-time employed individuals who report their jobs are so inflexible that they cannot attend primary care appointments during working hours. Using large, repeated, health-focused cross-sectional surveys for England, we map the geographic distribution of inflexible jobs, estimate the associations between job flexibility and health and healthcare utilisation, and exploit the large sample for subgroup analyses by gender, age, and deprivation.

METHODS

Study population

We used data from the national General Practice Patient Survey (GPPS) in England.[10] The survey is distributed by post to over two million people annually. It is sent to a sample of patients registered at each general practice in England and includes questions on job flexibility, health, primary healthcare use, socioeconomic characteristics, and the EQ-5D measure of health-related quality of life (HRQoL). We used data from six survey years, 2012 to 2017, when data on HRQoL was collected consistently using the five-level version of the EQ-5D (EQ-5D-5L).[11]

For peer review only - http://bmjopen.bmj.com/site/about/guidelines.xhtml

Procedures

If the respondent reported being in paid employment, they were asked: "*If you need to see a GP at your GP surgery during your typical working hours, can you take time away from your work to do this? Yes/No*".

The EQ-5D has five domains: 'mobility', 'self-care', 'usual activities', 'pain/discomfort', and 'anxiety/depression'. Individuals were asked to report their health today in each domain on a five-point ordinal scale, where (1) corresponds to no problems; (2) slight problems; (3) moderate problems; (4) severe problems and (5) extreme problems/unable to do activities. We analysed each domain separately and a composite utility score, obtained by mapping the 5-level to the 3-level version using the cross-walk tool described in van Hout et al.[12]

We also analysed utilisation of three types of healthcare services. The first two types were GP and nurse visits. The GPPS asks how recently the respondent has seen or spoken to (1) a GP and (2) a nurse at their GP practice, with five possible responses ranging from *"in the past three months"* to *"I have never seen a [GP/nurse] from my surgery"*. We recoded these responses to create intervals for the number of months since the respondent had seen a GP/nurse (see Supplementary Table 1). The third type of healthcare was use of out-of-hours primary care. The GPPS asks the question *"In the past 6 months, have you tried to call an out-of-hours GP service when [your usual] surgery was closed? Yes, for myself/ Yes, for someone else/ No"*. We considered only calls made for the respondent themself.

The GPPS contains information on the gender of each respondent, their age (in age bands: 18-24 years; 25-34 years; 35-44 years; 45-54 years; 55-64 years), their ethnicity (white; mixed; Asian; black; other), whether they had ever smoked, and whether they were a parent/guardian to a child aged 16 years and under.

England is divided into 326 geographic areas called local authority districts (LADs). At the LAD level, we obtained annual information on the share of the working population employed in each of the nine broad categories of Standard Occupational Classification (SOC)[13]: 1) managers, directors and senior officials; 2) professional; 3) associate professional and technical; 4) administrative and secretarial; 5) skilled trade; 6) caring, leisure and other service; 7) sales and customer services; 8) process, plant and machine operatives; and 9) elementary occupations. We obtained these variables via NOMIS: the UK Labour Market statistics portal [14] and merged them by LAD to the individual-level data.

For peer review only - http://bmjopen.bmj.com/site/about/guidelines.xhtml

Each LAD is further broken down into a collection of 32,844 smaller geographic areas called lower-level super output areas (LSOAs). LSOAs contain on average 1,500 residents. Levels of deprivation were calculated at LSOA level using the 2015 iteration of the Index of Multiple Deprivation (IMD).[15] We used deciles of area deprivation, where 1 is the least deprived decile and 10 is the most deprived decile.

We restricted the main analysis to individuals who reported working full time. We did this to avoid issues associated with part-time working. For example, we believe that part-time and full-time individuals face different choice sets when it comes to time allocation. As stated above, the main variable we are concerned with relates to an individual's ability to visit a GP during their *usual working hours*. This definition of usual working hours is likely to vary between full- and part-time employees. As a robustness check, we relaxed the assumption relating to full-time employment by including part-time workers in the estimation sample.

We also restricted the analysis to those within the statutory working-age range, 18-64 years. This is to minimise endogeneity generated by voluntary participation in work beyond state retirement age.

We retained observations with non-missing data for all outcomes and covariates. In the supplementary material we provide a flowchart which shows the number of observations dropped at each stage of data preparation (Supplementary Figure 1).

Statistical analyses

We calculated the percentage of GPPS respondents who reported having an inflexible job in each of the 326 LADs that existed as of 2017 in England. We used spmap [16] to create a heat map showing the percentage for each area. We compared this to a heat map of the mean EQ-5D utility score for GPPS respondents in each area.

We used linear regression to estimate the strength of association between HRQoL and having an inflexible job, adjusting for person characteristics, LSOA characteristics, local authority characteristics, and fixed effects for local authorities and survey years. We used interval regression[17] for the number of months since the respondent last saw (1) a GP or (2) a nurse. We estimated all models with robust standard errors, and used the weights provided with the survey data which correct for the GPPS sampling design and reduce the impact of non-response bias.

For peer review only - http://bmjopen.bmj.com/site/about/guidelines.xhtml

To investigate differences between subgroups, we estimated all models for men and women separately. We additionally estimated the strength of association between having an inflexible job and the EQ-5D utility score for subgroups of age and area deprivation decile.

Patient and public involvement

Patients or the public were not involved in the design of this study.

RESULTS

The estimation sample comprised 543,737 women and 629,488 men, aged 18-64 years and in full-time employment. Thirty-one percent of respondents reported that their job was inflexible. There were gender differences, with men reporting lower levels than women (28% compared to 34%; Table 1).

Panel (a) of Supplementary Figure 2 shows the geographic distribution of inflexible jobs. The areas with the highest concentration of inflexible jobs (darkest shading) were areas of the north-east, the north-west, west Midlands, and London. These areas are traditionally the areas with the highest levels of deprivation and the highest concentration of manual jobs. Supplementary Tables 2 and 3 provide greater detail on which local authorities had the highest and lowest percentages of respondents who reported inflexible jobs.

Panel (b) of Supplementary Figure 2 shows the geographic distribution of mean EQ-5D utility score. Darker areas indicate worse health, and these are present in both the north and the south. London has a high concentration of light-coloured areas which represent good average health. Excluding London, there was an apparent correlation between the two measures: areas with higher rates of inflexible jobs, on average, had lower levels of health. The raw individual level correlation of -0.053 between reporting an inflexible job and EQ-5D utility score confirms this.

The mean EQ-5D utility score for women was 0.88, which is slightly lower than the corresponding value of 0.89 for men (Table 1). The highest mean EQ-5D domain score was self-care (4.97/5). In descending order, the other mean domain scores were: mobility (4.85); usual activities (4.83); anxiety/depression (4.66); and pain/discomfort (4.47). All domains

For peer review only - http://bmjopen.bmj.com/site/about/guidelines.xhtml

were very similar across gender, except anxiety/depression, where men reported higher scores (which corresponds to lower levels of anxiety/depression) than women.

Table 1: Selected summary statistics for males and females

	Female		Male	
	**Mean**	**Std. Dev.**	**Mean**	**Std. Dev.**
Outcomes				
EQ-5D utility score	0.88	0.15	0.89	0.14
EQ-5D: mobility domain	4.86	0.46	4.84	0.49
EQ-5D: self-care domain	4.97	0.21	4.97	0.23
EQ-5D: usual activities domain	4.83	0.50	4.82	0.51
EQ-5D: pain/discomfort domain	4.47	0.76	4.48	0.74
EQ-5D: anxiety/depression domain	4.62	0.69	4.70	0.63
Months since last saw GP				
0 to 3	0.54	0.50	0.41	0.49
3 to 6	0.19	0.39	0.18	0.39
6 to 12	0.15	0.35	0.17	0.38
12 to 24	0.12	0.32	0.21	0.41
24+	0.01	0.10	0.02	0.14
Months since last saw nurse				
0 to 3	0.33	0.47	0.24	0.43
3 to 6	0.19	0.39	0.14	0.35
6 to 12	0.19	0.39	0.16	0.37
12 to 24	0.25	0.43	0.34	0.47
24+	0.04	0.19	0.12	0.32
Out-of-hours (1 if contacted in last 6 months)	0.03	0.17	0.02	0.14
Key variable				
Inflexible job	0.34	0.47	0.28	0.45
Individual level characteristics				
Parent	0.24	0.43	0.32	0.47
Aged 18-24 years	0.06	0.24	0.04	0.19
Aged 25-34 years	0.21	0.41	0.14	0.35
Aged 35-44 years	0.22	0.42	0.22	0.42
Aged 45-54 years	0.31	0.46	0.32	0.47
Aged 55-64 years	0.19	0.39	0.27	0.45
Ethnicity: white	0.87	0.34	0.85	0.35
Ethnicity: mixed	0.01	0.11	0.01	0.09
Ethnicity: Asian	0.06	0.24	0.08	0.28
Ethnicity: black	0.04	0.19	0.03	0.17
Ethnicity: other	0.02	0.13	0.03	0.16
Never smoked	0.62	0.49	0.55	0.5
Commuting time = 0 (work at home)	0.05	0.21	0.06	0.23
Local occupational composition				

For peer review only

SOC1: managers, directors and senior officials	10.44	3.22	10.39	3.18
SOC2: professional	19.93	5.54	19.67	5.42
SOC3: associate professional and technical	14.33	3.74	14.15	3.61
SOC4: administrative and secretarial	10.63	2.36	10.66	2.36
SOC5: skilled trade	10.19	3.20	10.35	3.15
SOC6: caring, leisure and other service	9.03	2.25	9.07	2.23
SOC7: sales and customer services	7.74	2.22	7.78	2.20
SOC8: process, plant and machine operatives	6.43	2.75	6.56	2.74
SOC9: elementary occupations	10.74	3.34	10.84	3.33
	N=543,737		N=629,488	

Data source: GPPS pooled data for 2012 to 2017; respondents in full-time employment; SOC = standard occupational classification.

For peer review only

The modal response was 'no problems' in each of the EQ-5D domains. This mode was much lower for the pain/discomfort domain (60% in level 5) compared to self-care (98% in level 5). Anxiety/depression also had a relatively low number of people in level 5 (75%).

32% of men and 24% of women were parents, and the modal age group was 45-54 years of age. The sample was predominantly white (87%), with 6% Asian, 4% black, 2% other and 1% mixed (Table 1).

There was a good spread of occupational classifications across the Local Authority Districts. The modal occupational classification was professional (SOC2; approximately 20%) and the classification with the least representation is process, plant and machine operatives (SOC8; 6.5%).

Individuals in the sample tended to have seen a GP more recently than a nurse (Table 1). 67% saw a GP in the last six months, compared to 46% who saw a nurse. 1% of individuals had never seen a GP in their practice, compared to 7% who had never seen a nurse.

Respondents living in more deprived areas were more likely to have an inflexible job compared to those living in less deprived areas (Table 2; columns (1) and (2)). Older respondents were less likely than younger respondents to report having an inflexible job.

Women whose jobs were inflexible had average EQ-5D utility scores that were 0.017 (95% confidence interval (CI) [0.016; 0.018]) lower than their counterparts with a flexible job (Table 2; column (3)). Men whose jobs were inflexible had average EQ-5D utility scores that were 0.016 (95% CI [0.015; 0.017]) lower than their counterparts with a flexible job (Table 2; column (4)). The standard deviation of the EQ-5D utility score was 0.15 for women and 0.14

For peer review only - http://bmjopen.bmj.com/site/about/guidelines.xhtml

for men (Table 1). This means that the reduction was about 11% of one standard deviation for both women and men.

Table 2: The effect of covariates on reporting an inflexible job and the effect of an inflexible job on the EQ-5D utility score

	Inflexible job		EQ-5D utility score	
	Females (1)	Males (2)	Females (3)	Males (4)
Inflexible job			-0.017 [-0.018; -0.016]	-0.016 [-0.017; -0.015]
Parent	-0.005 [-0.008; -0.001]	0.002 [-0.001; 0.006]	0.018 [0.017; 0.019]	0.011 [0.010; 0.012]
Aged 25-34 years [a]	-0.065 [-0.072; -0.057]	-0.061 [-0.070; -0.053]	0.001 [-0.001; 0.003]	-0.020 [-0.022; -0.017]
Aged 35-44 years [a]	-0.096 [-0.104; -0.089]	-0.089 [-0.097; -0.081]	-0.020 [-0.022; -0.018]	-0.038 [-0.040; -0.036]
Aged 45-54 years [a]	-0.106 [-0.112; -0.099]	-0.101 [-0.108; -0.093]	-0.039 [-0.041; -0.037]	-0.056 [-0.058; -0.054]
Aged 55-64 years [a]	-0.133 [-0.141; -0.126]	-0.130 [-0.138; -0.122]	-0.051 [-0.053; -0.049]	-0.072 [-0.074; -0.070]
Ethnicity: mixed [b]	0.003 [-0.011; 0.017]	0.003 [-0.013; 0.018]	-0.011 [-0.015; -0.006]	-0.003 [-0.008; 0.001]
Ethnicity: Asian [b]	0.003 [-0.004; 0.010]	0.023 [0.017; 0.029]	0.005 [0.003; 0.007]	0.007 [0.005; 0.009]
Ethnicity: black [b]	-0.034 [-0.043; -0.025]	-0.011 [-0.021; -0.002]	0.001 [-0.002; 0.003]	0.017 [0.015; 0.020]
Ethnicity: other [b]	-0.000 [-0.012; 0.012]	0.013 [0.003; 0.022]	-0.010 [-0.014; -0.006]	-0.005 [-0.009; -0.001]
Never smoked	0.012 [0.008; 0.015]	-0.025 [-0.028; -0.022]	0.026 [0.025; 0.027]	0.025 [0.024; 0.026]
Deprivation decile 1 (least deprived)	-0.009 [-0.016; -0.001]	-0.031 [-0.038; -0.024]	0.010 [0.008; 0.012]	0.011 [0.009; 0.013]
Deprivation decile 2	-0.013 [-0.021; -0.006]	-0.019 [-0.026; -0.013]	0.006 [0.004; 0.008]	0.008 [0.006; 0.010]
Deprivation decile 3	-0.010 [-0.018; -0.003]	-0.017 [-0.024; -0.011]	0.004 [0.002; 0.006]	0.006 [0.004; 0.008]
Deprivation decile 4	-0.007 [-0.014; 0.000]	-0.004 [-0.010; 0.003]	0.002 [-0.000; 0.004]	0.003 [0.001; 0.005]
Deprivation decile 6	0.004 [-0.004; 0.011]	0.007 [0.000; 0.014]	-0.004 [-0.006; -0.002]	-0.003 [-0.005; -0.001]
Deprivation decile 7	0.008 [0.001; 0.015]	0.018 [0.011; 0.024]	-0.010 [-0.012; -0.007]	-0.008 [-0.010; -0.006]
Deprivation decile 8	0.014 [0.007; 0.022]	0.025 [0.018; 0.032]	-0.012 [-0.014; -0.010]	-0.009 [-0.011; -0.007]

For peer review only

Deprivation decile 9	0.028 [0.021; 0.036]	0.041 [0.034; 0.048]	-0.015 [-0.018; -0.013]	-0.013 [-0.015; -0.011]
Deprivation decile 10 (most deprived)	0.041 [0.033; 0.049]	0.054 [0.046; 0.061]	-0.022 [-0.025; -0.020]	-0.017 [-0.019; -0.015]
Other controls [d]	Yes	Yes	Yes	Yes
Observations	543,737	629,488	543,737	629,488

95% confidence intervals in parentheses.

[a] The reference category for age group is 16-24 years.

[b] The reference category for ethnic group is white.

[c] The reference category for deprivation is decile 5.

[d] All models additionally include the proportion of the workforce who work in each SOC group within the respondent's Local Authority District of residence, and fixed effects for survey year and Local Authority District of residence.

For the five separate domains of the EQ-5D (Table 3), the effects for men were larger in absolute magnitude in the domains of mobility, self-care, usual activities, and pain/discomfort. However, for the anxiety/depression domain, the effect is larger for women than men. For men and women, the biggest reductions were in the pain/discomfort domain, followed by anxiety/depression, then usual activities, then mobility, and finally self-care.

There is both a deprivation and age gradient on the effect of an inflexible job on health (Figure 1). The negative effect of having an inflexible job on health was larger in magnitude for people living in a more deprived area compared to those living in a less deprived area, and larger in magnitude for older people compared to younger people.

Having an inflexible job was associated with less recent utilisation of both GPs and nurses (Table 4). For women with an inflexible job, it had been an additional 0.178 (95% CI [0.145; 0.212]) months since they had seen a GP at their surgery, compared to women with a flexible job. The magnitude of this effect was greater for nurse appointments than GP appointments; for women with an inflexible job it had been an additional 0.493 (95% CI [0.445; 0.541]) months since they had seen a nurse at their GP surgery, compared to women with a flexible job. For men with an inflexible job, it had been an additional 0.146 (95% CI [0.010; 0.192]) months since they last saw a GP at their surgery, and an additional 0.554 (95% CI [0.487; 0.621]) months since they last saw a nurse at their GP surgery, compared to men with a flexible job. Conversely, individuals with inflexible jobs made more use of 'out-of-hours' GP appointments than their counterparts with flexible jobs.

For peer review only - http://bmjopen.bmj.com/site/about/guidelines.xhtml

Table 3: The effects of an inflexible job on the EQ-5D domain levels

	Mobility		Self-care		Usual activities		Pain/discomfort		Anxiety/depression	
	Females	Males	Females	Males	Females	Males	Females	Males	Females	Males
Inflexible job	-0.019	-0.025	-0.004	-0.005	-0.032	-0.035	-0.084	-0.088	-0.059	-0.038
	[-0.022; -0.016]	[-0.028; -0.022]	[-0.005; -0.002]	[-0.007; -0.004]	[-0.035; -0.029]	[-0.038; -0.032]	[-0.089; -0.079]	[-0.093; -0.083]	[-0.065; -0.054]	[-0.043; -0.034]
Observations	543,737	629,488	543,737	629,488	543,737	629,488	543,737	629,488	543,737	629,488

95% confidence intervals in parentheses.
All models additionally include indicators for: parental status, ten-year age group, ethnicity, whether the respondent has ever smoked, index of multiple deprivation decile; in addition to the proportion of the workforce who work in each SOC group within the respondent's Local Authority District of residence, and fixed effects for survey year and Local Authority District of residence.
All models estimated using ordinary least squares regression.

Table 4: The effects of an inflexible job on health care utilisation

	Months since last saw GP		Months since last saw nurse		Tried to call out-of-hours service in past 6 months	
	Females (1)	Males (2)	Females (3)	Males (4)	Females (5)	Males (6)
Inflexible job	0.178	0.146	0.493	0.554	0.007	0.005
	[0.145; 0.212]	[0.010; 0.192]	[0.445; 0.541]	[0.487; 0.621]	[0.006; 0.009]	[0.004; 0.007]
N	543,737	629,488	543,737	629,488	543,737	629,488

95% confidence intervals in parentheses.
All models additionally include indicators for: parental status, ten-year age group, ethnicity, whether the respondent has ever smoked, index of multiple deprivation decile; in addition to the proportion of the workforce who work in each SOC group within the respondent's Local Authority District of residence, and fixed effects for survey year and Local Authority District of residence.
Models (1) to (4) estimated using interval regression; Models (5) and (6) estimated using ordinary least squares regression.

For peer review only - http://bmjopen.bmj.com/site/about/guidelines.xhtml

The negative effect of an inflexible job on health remains when part time workers are included in the estimation sample (Supplementary Table 4). The main coefficients of interest (on 'inflexible job') are almost identical in magnitude and statistical significance. Part-time workers, on average, reported lower levels of HRQoL. This could be attributable, at least in part, to selective sorting into part-time jobs by people with lower level of HRQoL.

DISCUSSION

We found that individuals who had inflexible jobs were less likely to be in good health and the result was common to men and women. Our subgroup analyses of different age groups and people living in areas of differing deprivation suggest that the most affected groups were older aged people and those living in the most deprived areas. Older aged people were less likely to experience inflexible jobs, but their health was more affected if they did, compared to younger people. People living in more deprived areas were both more likely to have an inflexible job, and their health was more affected than those in less deprived areas.

When considering subdomains of health separately, we found that men were more affected in domains that more closely align to physical health (pain/discomfort and usual activities) whereas women were more affected in the anxiety/depression domain. This is a similar finding to Moen et al.[6] which found that women especially benefitted from a reduction in perceived stress and psychological stress when randomised into an intervention with greater work-time control.

We found that individuals with inflexible jobs made fewer visits to see their GP or a nurse compared to individuals with flexible jobs. As would perhaps be expected, individuals with inflexible jobs used out-of-hours services more often than individuals with flexible jobs, but still overall went to see their GP/nurse less frequently. It is possible that the lower opportunity cost of time for workers who were able to consume healthcare during working hours contributed to their more frequent visits to their GP.

We contribute to the evidence base on the relationship between work flexibility and health, which is likely to become an increasingly important consideration due to changes in working norms induced by the coronavirus pandemic.[18–20] The occupations which have seen increased home working during the pandemic (typically those where work can be carried out

For peer review only - http://bmjopen.bmj.com/site/about/guidelines.xhtml

using IT workarounds) are disproportionately those which are well paid [21] and require higher qualifications and more experience.[18] An increase in flexible working in these occupations could make already "good" jobs even better, widening the gap between "good" jobs and "bad" jobs. This could contribute to widening inequalities in health.

Additionally, we add to the very limited evidence base on the relationship between work flexibility and health care utilisation. If having an inflexible job represents an additional barrier to utilising routine health care, this may be one factor contributing to negative health effects of inflexible jobs. Typically, the onus has been put on the health care system to accommodate inflexible jobs by offering "out-of-hours" appointments, in the evenings and on weekends. However, perhaps there should be a greater responsibility for employers to ensure that systems are in place so that their employees are able to attend routine health care appointments during usual working hours, when they need to.

Limitations

There are well-known empirical barriers to establishing a causal relationship between job characteristics and health. In particular, people with certain unobservable characteristics have a tendency to self-select into certain occupations. Part of the association we found between inflexible jobs and health is likely to be due to self-selection and/or reverse causality.

The people we identified as having inflexible jobs may also have been more likely to have jobs with other "bad" characteristics such as a highly stressful work environment or a casual employment contract. We did not control for other bad job characteristics at an individual level, as this information is not included in the GPPS. However, we did control for the occupational composition of the area in which each respondent was located, which provided some adjustment for the characteristics of different job groups.

We identified people who had an inflexible job as those who responded that they were unable to go to see their GP in their usual working hours. There are, in fact, two possible types of job that would result in giving this response: those who worked "normal hours" (eg. 9am to 5pm) but were unable to take time off during those hours to visit their GP; and those who worked unsociable hours, so could not visit their GP during their working hours because the GP service was not open. Both of these job features are less desirable and may have a harmful effect on health.

For peer review only - http://bmjopen.bmj.com/site/about/guidelines.xhtml

The source of individual level data we used is from a survey intended to gather information on patients' experience of general practice (GPPS). It is possible that people were more likely to respond to the survey if they had used their general practice recently. This may affect generalisability to the general population.

Conclusion

This is the first study to examine the relationship between job flexibility and health and health care utilisation in a large dataset. We found that having an inflexible job – defined as not being able to take time away from work during usual working hours to visit a medical professional – was associated with reduced HRQoL and less recent visits to see a GP or nurse. This relationship was consistent for both men and women.

Individuals living in more deprived areas were more likely to report having an inflexible job, and this inflexible job was more likely to have a negative impact on their health. This suggests that flexible working may have the potential to either exacerbate or reduce existing inequalities in health, depending on how it is distributed throughout the working population.

DECLARATIONS

Ethics approval and consent to participate

Not applicable

Consent for publication

Not applicable

Availability of data and materials

The General Practice Patient Survey data that support the findings of this study are available from NHS England but restrictions apply to the availability of these data, which were used under license for the current study, and so are not publicly available.

Competing interests

The authors declare that they have no competing interests.

For peer review only - http://bmjopen.bmj.com/site/about/guidelines.xhtml

Funding

Charlie Moss received funding from a National Institute for Health Research (NIHR) Research Methods Fellowship [RM-FI-2017-08-018]. Luke Munford was supported by the Medical Research Council, through a Skills Development Fellowship [MR/N015126/1]. Matt Sutton was an NIHR Senior Investigator. The views expressed are those of the authors and not necessarily those of the MRC, the NIHR or the Department of Health and Social Care.

Authors' contributions

MS had the original idea for the study. MS and LM designed the analysis. CM carried out the analysis. CM, LM and MS contributed substantially to the interpretation of results and the writing of the manuscript.

Acknowledgements

We are grateful for comments and suggestions received from colleagues at HOPE and the Melbourne Institute: Applied Economic and Social Research. Additional thanks to Nordic Health Economists' Study Group 2019 conference attendees for a useful discussion of a previous version.

For peer review only

REFERENCES

1 Costa PG, Sartori S, Åkerstedt T. Influence of Flexibility and Variability of Working Hours on Health and Well-Being. *Chronobiology International* 2006;**23**:1125–37. doi:10.1080/07420520601087491

2 Joyce K, Pabayo R, Critchley JA, *et al.* Flexible working conditions and their effects on employee health and wellbeing. *Cochrane Database of Systematic Reviews* Published Online First: 2010. doi:10.1002/14651858.CD008009.pub2

3 Nijp HH, Beckers DG, Geurts SA, *et al.* Systematic review on the association between employee worktime control and work-non-work balance, health and well-being, and job-related outcomes. *Scandinavian Journal of Work, Environment & Health* 2012;**38**:299–313.

4 Brauner C, Wöhrmann AM, Frank K, *et al.* Health and work-life balance across types of work schedules: A latent class analysis. *Applied Ergonomics* 2019;**81**:102906. doi:10.1016/j.apergo.2019.102906

5 Albrecht SC, Kecklund G, Rajaleid K, *et al.* The longitudinal relationship between control over working hours and depressive symptoms: Results from SLOSH, a

For peer review only - http://bmjopen.bmj.com/site/about/guidelines.xhtml

population-based cohort study. *Journal of Affective Disorders* 2017;**215**:143–51. doi:10.1016/j.jad.2017.03.010

6 Moen P, Kelly EL, Fan W, *et al.* Does a Flexibility/Support Organizational Initiative Improve High-Tech Employees' Well-Being? Evidence from the Work, Family, and Health Network. *Am Sociol Rev* 2016;**81**:134–64. doi:10.1177/0003122415622391

7 Ropponen A, Känsälä M, Rantanen J, *et al.* Organizational Initiatives for Promoting Employee Work-Life Reconciliation Over the Life Course. A Systematic Review of Intervention Studies. *Nordic Journal of Working Life Studies* 2016;**6**. doi:10.19154/njwls.v6i3.5529

8 Nätti J, Oinas T, Anttila T. Time pressure, working time control and long-term sickness absence. *Occup Environ Med* 2015;**72**:265–70. doi:10.1136/oemed-2014-102435

9 Office for National Statistics. Living longer: impact of working from home on older workers. 2021. https://www.ons.gov.uk/peoplepopulationandcommunity/birthsdeathsandmarriages/ageing/articles/livinglongerimpactofworkingfromhomeonolderworkers/2021-08-25 (accessed 4 Nov 2021).

10 GP Patient Survey. About the survey. 2020.https://www.gp-patient.co.uk/About (accessed 19 Aug 2020).

11 Brooks R. EuroQol: the current state of play. *Health Policy* 1996;**37**:53–72. doi:10.1016/0168-8510(96)00822-6

12 van Hout B, Janssen MF, Feng Y-S, *et al.* Interim Scoring for the EQ-5D-5L: Mapping the EQ-5D-5L to EQ-5D-3L Value Sets. *Value in Health* 2012;**15**:708–15. doi:10.1016/j.jval.2012.02.008

13 Office for National Statistics. SOC2010 volume 1: structure and descriptions of unit groups. 2010.https://www.ons.gov.uk/methodology/classificationsandstandards/standardoccupationalclassificationsoc/soc2010/soc2010volume1structureanddescriptionsofunitgroups (accessed 24 Apr 2020).

14 Office for National Statistics. Nomis - Official Labour Market Statistics. 2020.https://www.nomisweb.co.uk/ (accessed 4 Nov 2020).

15 Office for National Statistics. English indices of deprivation 2015. GOV.UK. 2015.https://www.gov.uk/government/statistics/english-indices-of-deprivation-2015 (accessed 4 Nov 2020).

16 Pisati M. *SPMAP: Stata module to visualize spatial data*. 2018. https://econpapers.repec.org/software/bocbocode/s456812.htm (accessed 23 Aug 2020).

17 Stewart MB. On Least Squares Estimation when the Dependent Variable is Grouped. *The Review of Economic Studies* 1983;**50**:737–53. doi:10.2307/2297773

18 Office for National Statistics. Coronavirus and homeworking in the UK. 2020.https://www.ons.gov.uk/employmentandlabourmarket/peopleinwork/employmentan

For peer review only - http://bmjopen.bmj.com/site/about/guidelines.xhtml

demployeetypes/bulletins/coronavirusandhomeworkingintheuk/april2020 (accessed 5 Nov 2020).

19 Bloom N. How working from home works out. Stanford Institute for Economic Policy Research 2020. https://siepr.stanford.edu/sites/default/files/publications/SIEPR%20Policy%20Brief%20June%202020%20v04.pdf (accessed 11 May 2020).

20 The Economist. Covid-19 has forced a radical shift in working habits. *The Economist* 2020.https://www.economist.com/briefing/2020/09/12/covid-19-has-forced-a-radical-shift-in-working-habits (accessed 5 Nov 2020).

21 Dingel JI, Neiman B. How Many Jobs Can be Done at Home? National Bureau of Economic Research 2020. doi:10.3386/w26948

For peer review only

Figure legend

Figure 1: The effect of an inflexible job on EQ-5D utility score for subgroups of age and deprivation

Notes:

Bars show 95% confidence intervals.

All models additionally include indicators for: parental status, ten-year age group, ethnicity, whether the respondent has ever smoked, index of multiple deprivation decile; in addition to the proportion of the workforce who work in each SOC group within the respondent's Local Authority District of residence, and fixed effects for survey year and Local Authority District of residence.

For peer review only - http://bmjopen.bmj.com/site/about/guidelines.xhtml

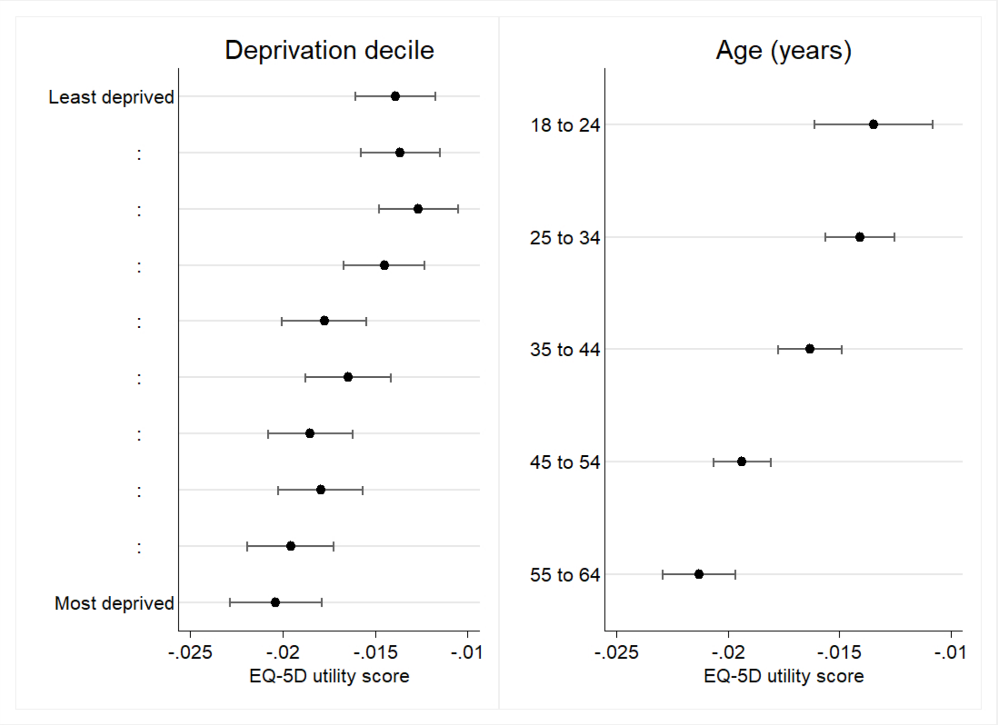

388x282mm (72 x 72 DPI)

SUPPLEMENTARY MATERIAL

Supplementary Table 1: Possible responses to survey questions "when did you last see or speak to a GP [nurse] from your GP surgery?" and corresponding intervals

Response	Months since last saw/spoke to GP/nurse
In the past 3 months	0 to 3
Between 3 and 6 months ago	3 to 6
Between 6 and 12 months ago	6 to 12
More than 12 months ago	12 to 24
I have never seen a GP from my GP surgery	24+

Data source: General Practice Patient Survey, Questions 1 and 2

For peer review only

For peer review only - http://bmjopen.bmj.com/site/about/guidelines.xhtml

Supplementary Table 2: Local Authority Districts with lowest percentage of respondents reporting an inflexible job

Local Authority District	% reporting inflexible job	N
Isles of Scilly	13.1	175
City of London	19.2	240
Cambridge	19.4	2,847
Ryedale	20.7	1,282
Oxford	21.0	4,000
Melton	21.0	932
South Lakeland	21.3	2,987
South Cambridgeshire	21.8	4,509
West Somerset	21.9	987
Vale of White Horse	22.1	2,676
Cotswold	22.3	2,312
Rutland	22.4	706
Wokingham	22.8	3,156
South Oxfordshire	22.9	3,039
South Somerset	22.9	4,517
Stroud	22.9	3,377
Derbyshire Dales	23.1	1,969
West Oxfordshire	23.2	2,498
Wiltshire	23.3	10,662
Eden	23.3	1,813

Data source: GPPS respondents in full-time employment, 2012-2017

For peer review only

Supplementary Table 3: Local Authority Districts with highest percentage of respondents reporting an inflexible job

Local Authority District	% reporting inflexible job	N
Barking and Dagenham	40.7	6,772
Newham	38.3	9,043
Knowsley	37.8	4,237
Thurrock	37.6	5,690
Barnsley	37.4	5,560
Hillingdon	36.8	9,755
Basildon	36.8	5,752
Dartford	36.6	2,888
Havering	36.5	8,012
Luton	36.5	5,089
Sandwell	36.5	9,165
St. Helens	36.4	5,505
Bolsover	36.4	73
Medway	36.3	9,291
Mansfield	36.2	2,273
Crawley	36.1	2,499
Gravesham	36.1	2,506
Rotherham	36.1	5,677
Wakefield	36.1	6,635
Redcar and Cleveland	35.9	3,176

Data source: GPPS respondents in full-time employment, 2012-2017

For peer review only

Supplementary Table 4: The effect of covariates on reporting an inflexible job and the effect of an inflexible job on the EQ-5D utility score; full-time and part-time workers

	Inflexible job		EQ-5D	
	Females (1)	Males (2)	Females (3)	Males (4)
Inflexible job			-0.018 [-0.019; -0.017]	-0.016 [-0.017; -0.015]
Part-time	0.027 [0.025; 0.030]	-0.017 [-0.022; -0.013]	-0.016 [-0.017; -0.016]	-0.038 [-0.040; -0.036]
Parent	-0.005 [-0.008; -0.002]	0.002 [-0.001; 0.006]	0.024 [0.023; 0.025]	0.011 [0.010; 0.012]
Aged 25-34 years [a]	-0.055 [-0.061; -0.049]	-0.060 [-0.068; -0.052]	-0.000 [-0.002; 0.001]	-0.023 [-0.026; -0.021]
Aged 35-44 years [a]	-0.076 [-0.082; -0.070]	-0.087 [-0.095; -0.080]	-0.021 [-0.022; -0.019]	-0.043 [-0.045; -0.041]
Aged 45-54 years [a]	-0.086 [-0.092; -0.080]	-0.100 [-0.107; -0.092]	-0.039 [-0.041; -0.038]	-0.061 [-0.063; -0.059]
Aged 55-64 years [a]	-0.117 [-0.123; -0.111]	-0.131 [-0.138; -0.123]	-0.048 [-0.049; -0.046]	-0.074 [-0.076; -0.072]
Ethnicity: mixed [b]	0.001 [-0.011; 0.013]	0.008 [-0.007; 0.023]	-0.014 [-0.018; -0.010]	-0.006 [-0.010; -0.001]
Ethnicity: Asian [b]	-0.003 [-0.009; 0.003]	0.017 [0.012; 0.023]	0.001 [-0.001; 0.002]	0.008 [0.006; 0.010]
Ethnicity: black [b]	-0.038 [-0.045; -0.030]	-0.010 [-0.019; -0.002]	-0.002 [-0.004; 0.001]	0.020 [0.017; 0.022]
Ethnicity: other [b]	-0.017 [-0.027; -0.008]	0.006 [-0.002; 0.015]	-0.014 [-0.017; -0.010]	-0.004 [-0.008; -0.001]
Never smoked	0.009 [0.006; 0.012]	-0.023 [-0.026; -0.020]	0.027 [0.027; 0.028]	0.026 [0.025; 0.027]
Deprivation decile 1 (least deprived)	-0.006 [-0.012; -0.000]	-0.029 [-0.036; -0.023]	0.012 [0.011; 0.014]	0.012 [0.010; 0.014]
Deprivation decile 2	-0.010 [-0.016; -0.004]	-0.019 [-0.025; -0.013]	0.008 [0.006; 0.010]	0.009 [0.007; 0.010]
Deprivation decile 3	-0.007 [-0.013; -0.001]	-0.017 [-0.023; -0.011]	0.006 [0.004; 0.007]	0.007 [0.005; 0.009]
Deprivation decile 4	-0.005 [-0.011; 0.000]	-0.005 [-0.011; 0.002]	0.002 [0.000; 0.004]	0.003 [0.001; 0.005]
Deprivation decile 6	0.007 [0.002; 0.013]	0.008 [0.001; 0.014]	-0.005 [-0.006; -0.003]	-0.003 [-0.005; -0.001]
Deprivation decile 7	0.009 [0.003; 0.014]	0.017 [0.011; 0.024]	-0.010 [-0.011; -0.008]	-0.007 [-0.009; -0.005]
Deprivation decile 8	0.013 [0.008; 0.019]	0.024 [0.017; 0.030]	-0.014 [-0.016; -0.013]	-0.009 [-0.012; -0.007]
Deprivation decile 9	0.026 [0.020; 0.032]	0.039 [0.032; 0.045]	-0.018 [-0.020; -0.016]	-0.013 [-0.015; -0.010]

For peer review only - http://bmjopen.bmj.com/site/about/guidelines.xhtml

Deprivation decile 10 (most deprived)	0.036 [0.030; 0.042]	0.049 [0.042; 0.056]	-0.025 [-0.027; -0.023]	-0.017 [-0.019; -0.015]
Other controls [d]	Yes	Yes	Yes	Yes
Observations	866,634	706,390	866,634	706,390

95% confidence intervals in parentheses.

All models are estimated using ordinary least squares regression;

[a] The reference category for age group is 16-24 years;

[b] The reference category for ethnic group is white;

[c] The reference category for deprivation is decile 5;

[d] All models additionally include the proportion of the workforce who work in each SOC group within the respondent's Local Authority District of residence, and fixed effects for survey year and Local Authority District of residence.

For peer review only

For peer review only - http://bmjopen.bmj.com/site/about/guidelines.xhtml

Supplementary Figure 1: Flowchart of observations dropped at each stage of data preparation

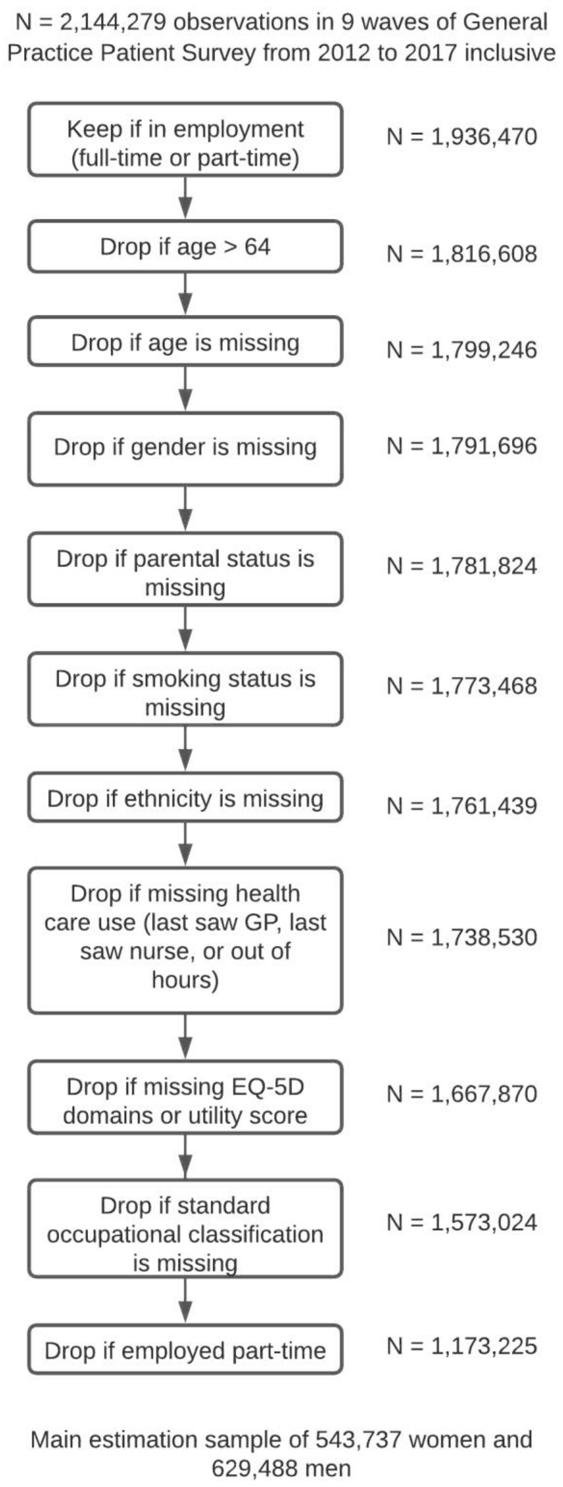

Supplementary Figure 2: Geographic distribution of inflexible jobs and health-related quality of life in England

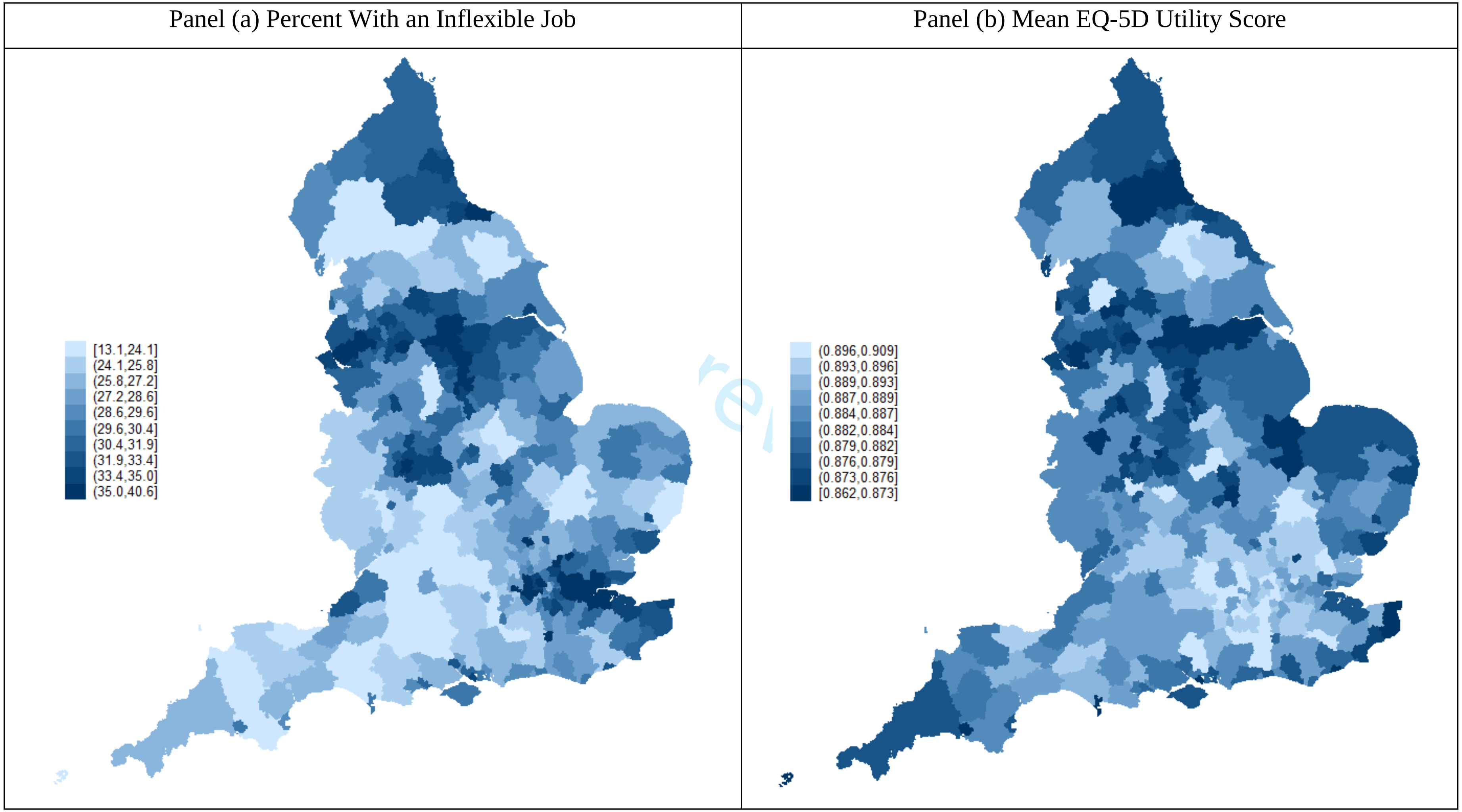

Data source: 1,346,001 General Practice Patient Survey respondents in full-time employment who have complete data for questions relating to job flexibility and EQ-5D utility score; pooled data from 2012-2017; respondents who report inability to visit their GP during working hours are classified as having an inflexible job; averages for 326 local authority districts; maps created using spmap (Pisati, 2018); deciles generated within spmap

STROBE Statement—Checklist of items that should be included in reports of ***cross-sectional studies***

	Item No	Recommendation	Where/how we have done this	Page number (main text word doc)
**Title and abstract**	1	(*a*) Indicate the study's design with a commonly used term in the title or the abstract	We included the term "retrospective cross-sectional study" in the title	1
		(*b*) Provide in the abstract an informative and balanced summary of what was done and what was found	We have done this	2
**Introduction**				
Background/rationale	2	Explain the scientific background and rationale for the investigation being reported	Done in paragraphs 2 and 3 of introduction	4
Objectives	3	State specific objectives, including any prespecified hypotheses	Third and final paragraph of introduction	4
**Methods**				
Study design	4	Present key elements of study design early in the paper	We have generally provided a lot of detail about the data we have used	4/5/6
Setting	5	Describe the setting, locations, and relevant dates, including periods of recruitment, exposure, follow-up, and data collection	We have done this in the "study population" subsection	4
Participants	6	(*a*) Give the eligibility criteria, and the sources and methods of selection of participants	We describe how the survey is distributed to respondents by GPPS, and we state our inclusion criteria, which is respondents who are aged between 18 and 64 and are in full time employment	4 and 6
Variables	7	Clearly define all outcomes, exposures, predictors, potential confounders, and effect modifiers. Give diagnostic criteria, if applicable	We provide detail of all covariates and outcomes included in our analyses	5 and 6
Data sources/ measurement	8*	For each variable of interest, give sources of data and details of methods of assessment (measurement). Describe comparability of assessment methods if there is more than one group	We give data sources for all variables, and background information relating to the EQ-5D-5L	5 and 6
Bias	9	Describe any efforts to address	In the methods section, we state	6

For peer review only - http://bmjopen.bmj.com/site/about/guidelines.xhtml

		potential sources of bias	that we only included employees aged 18 to 64 in our main analyses to minimise endogeneity generated by voluntary participation in the workforce beyond retirement age. We also state the covariates that we adjust for in the regression analyses.	
Study size	10	Explain how the study size was arrived at	We state our sample selection criteria in terms of age and employment status, and we provide a flowchart in the supplementary material which shows how we arrived at the sample size	6 and Supplementary Material p6
Quantitative variables	11	Explain how quantitative variables were handled in the analyses. If applicable, describe which groupings were chosen and why	We have described this in the methods section	5 and 6
Statistical methods	12	(*a*) Describe all statistical methods, including those used to control for confounding	In the "statistical analyses" subsection, we state that we used linear regression for most outcomes, except for two outcomes where we used interval regression	6
		(*b*) Describe any methods used to examine subgroups and interactions	We state in the "statistical analyses" subsection that we present all results split into subgroups for men and women, and we also present the results for the main outcome by subgroups of deprivation decile	7
		(*c*) Explain how missing data were addressed	In the "procedures" subsection we state that we retained observations with no missing data for covariates, and we provide a flowchart in the supplementary material	6 and Supplementary Material p6
		(*d*) If applicable, describe analytical methods taking account of sampling strategy	We state that we use the weights provided with the survey data	6
		(*e*) Describe any sensitivity analyses	We describe the sensitivity analysis where we include part-time employees in the sample	6
**Results**				
Participants	13*	(a) Report numbers of individuals at each stage of	We report the number of individuals that drop out of the	Supplementary Material p6

For peer review only - http://bmjopen.bmj.com/site/about/guidelines.xhtml

		study—eg numbers potentially eligible, examined for eligibility, confirmed eligible, included in the study, completing follow-up, and analysed	sample at each stage of data preparation in the supplementary material	
		(b) Give reasons for non-participation at each stage	See below	Supplementary Material p6
		(c) Consider use of a flow diagram	We provide a flowchart in the supplementary material	Supplementary Material p6
Descriptive data	14*	(a) Give characteristics of study participants (eg demographic, clinical, social) and information on exposures and potential confounders	In Table 1	8 and 9
		(b) Indicate number of participants with missing data for each variable of interest	This can be seen in the flowchart	Supplementary Material p6
Outcome data	15*	Report numbers of outcome events or summary measures	Table 1	8 and 9
Main results	16	(*a*) Give unadjusted estimates and, if applicable, confounder-adjusted estimates and their precision (eg, 95% confidence interval). Make clear which confounders were adjusted for and why they were included	We provide confidence intervals for all estimates	10-12
		(*b*) Report category boundaries when continuous variables were categorized	N/A – we did not categorise any continuous variables	
		(*c*) If relevant, consider translating estimates of relative risk into absolute risk for a meaningful time period	N/A	
Other analyses	17	Report other analyses done—eg analyses of subgroups and interactions, and sensitivity analyses	We report analyses for subgroups of gender, age, and deprivation, and sensitivity analysis where we include part-time employees	Figure 1 and Supplementary Material p4-5
**Discussion**				
Key results	18	Summarise key results with reference to study objectives	Discussion paragraphs 1-3	13
Limitations	19	Discuss limitations of the study, taking into account sources of potential bias or imprecision. Discuss both direction and magnitude of any potential bias	We include a "limitations" subsection with a range of considerations	14
Interpretation	20	Give a cautious overall interpretation of results	We do this in the "conclusion" subsection	15

For peer review only - http://bmjopen.bmj.com/site/about/guidelines.xhtml

		considering objectives, limitations, multiplicity of analyses, results from similar studies, and other relevant evidence		
Generalisability	21	Discuss the generalisability (external validity) of the study results	We do this in the "limitations" subsection	14
**Other information**				
Funding	22	Give the source of funding and the role of the funders for the present study and, if applicable, for the original study on which the present article is based	We include a funding statement	16

*Give information separately for exposed and unexposed groups.

Note: An Explanation and Elaboration article discusses each checklist item and gives methodological background and published examples of transparent reporting. The STROBE checklist is best used in conjunction with this article (freely available on the Web sites of PLoS Medicine at http://www.plosmedicine.org/, Annals of Internal Medicine at http://www.annals.org/, and Epidemiology at http://www.epidem.com/). Information on the STROBE Initiative is available at www.strobe-statement.org.

For peer review only

For peer review only - http://bmjopen.bmj.com/site/about/guidelines.xhtml

BMJ Open

Associations between inflexible job conditions, health and healthcare utilisation in England: retrospective cross-sectional study

Journal:	*BMJ Open*
Manuscript ID	bmjopen-2022-062942.R1
Article Type:	Original research
Date Submitted by the Author:	21-Oct-2022
Complete List of Authors:	Moss, Charlie; The University of Manchester, Health Organisation, Policy and Economics (HOPE), Centre for Primary Care and Health Services Research, School of Health Sciences<br>Munford, Luke; The University of Manchester, Health Organisation, Policy and Economics (HOPE), Centre for Primary Care and Health Services Research, School of Health Sciences<br>Sutton, Matt; The University of Manchester, Health Organisation, Policy and Economics (HOPE), Centre for Primary Care and Health Services Research, School of Health Sciences; The University of Melbourne, Melbourne Institute: Applied Economic and Social Research
**Primary Subject Heading**:	Public health
Secondary Subject Heading:	Occupational and environmental medicine
Keywords:	PUBLIC HEALTH, OCCUPATIONAL & INDUSTRIAL MEDICINE, PRIMARY CARE, SOCIAL MEDICINE

SCHOLARONE™
Manuscripts

I, the Submitting Author has the right to grant and does grant on behalf of all authors of the Work (as defined in the below author licence), an exclusive licence and/or a non-exclusive licence for contributions from authors who are: i) UK Crown employees; ii) where BMJ has agreed a CC-BY licence shall apply, and/or iii) in accordance with the terms applicable for US Federal Government officers or employees acting as part of their official duties; on a worldwide, perpetual, irrevocable, royalty-free basis to BMJ Publishing Group Ltd ("BMJ") its licensees and where the relevant Journal is co-owned by BMJ to the co-owners of the Journal, to publish the Work in this journal and any other BMJ products and to exploit all rights, as set out in our licence.

The Submitting Author accepts and understands that any supply made under these terms is made by BMJ to the Submitting Author unless you are acting as an employee on behalf of your employer or a postgraduate student of an affiliated institution which is paying any applicable article publishing charge ("APC") for Open Access articles. Where the Submitting Author wishes to make the Work available on an Open Access basis (and intends to pay the relevant APC), the terms of reuse of such Open Access shall be governed by a Creative Commons licence – details of these licences and which Creative Commons licence will apply to this Work are set out in our licence referred to above.

Other than as permitted in any relevant BMJ Author's Self Archiving Policies, I confirm this Work has not been accepted for publication elsewhere, is not being considered for publication elsewhere and does not duplicate material already published. I confirm all authors consent to publication of this Work and authorise the granting of this licence.

For peer review only

Associations between inflexible job conditions, health and healthcare utilisation in England: retrospective cross-sectional study

Charlie Moss,[1] Luke Munford,[1] Matt Sutton,[1,2]

[1] Health Organisation, Policy and Economics (HOPE), Centre for Primary Care and Health Services Research, School of Health Sciences, The University of Manchester, Manchester, UK

[2] Melbourne Institute: Applied Economic and Social Research, The University of Melbourne, Melbourne, Australia

Corresponding author: Charlie Moss, 6.25 Williamson Building, The University of Manchester, Oxford Road, Manchester, M13 9PL, UK; charlie.moss@manchester.ac.uk

For peer review only

For peer review only - http://bmjopen.bmj.com/site/about/guidelines.xhtml

ABSTRACT

Objectives: To estimate the strength of association between having an inflexible job and health-related quality of life and health care utilisation; and to explore heterogeneity in the effects by gender, age and area-level deprivation.

Design: Retrospective cross-sectional study.

Setting: Seven waves of the English General Practice Patient Survey between 2012 and 2017.

Participants: 1,232,884 people aged 16 to 64 and in full-time employment.

Methods: We measured job inflexibility by inability to take time away from work during usual working hours to seek medical care. We used regression analyses to estimate the strength of association between outcomes and having an inflexible job, adjusting for person and area-level characteristics.

Primary and secondary outcome measures: Health-related quality of life (EQ-5D-5L); number of months since the respondent last saw a GP or nurse; use of out-of-hours general practice in the past six months.

Results: One-third of respondents reported job inflexibility. The probability of job inflexibility was higher at younger ages and in more deprived areas. Job inflexibility was associated with lower EQ-5D-5L utility scores of 0.017 (95% confidence interval (CI) [0.016; 0.018]) for women and 0.016 (95% CI [0.015; 0.017]) for men. Women were more affected than men in the mental health domain. The reduction in health-related quality of life associated with having an inflexible job was greater for employees who were older or lived in more deprived areas. Having an inflexible job was associated with a longer time since the last visit to their GP of 0.234 (95% CI [0.201; 0.268]) months for women and 0.199 (95% CI [0.152; 0.183]) months for men.

Conclusions: Inequalities in the prevalence of inflexible jobs contribute to inequalities in health. One mechanism may be through reduced access to healthcare. Policymakers and employers should ensure that all employees have sufficient job flexibility to protect their health.

For peer review only

Keywords: work; health; health care; job flexibility

Strengths and limitations of this study

- Large sample of 1,232,884 survey respondents.
- We were able to use the EQ-5D-5L instrument which is a validated measure of health-related quality of life.
- The GPPS is a repeated cross-sectional survey. This makes it difficult to establish causality.
- The measure of job flexibility may not fully capture other time constraints that are unrelated to health care utilisation during working hours.

For peer review only - http://bmjopen.bmj.com/site/about/guidelines.xhtml

INTRODUCTION

Employee-oriented work flexibility refers to workers' discretion over some aspect of their job, such as location or hours of work. Examples include "flexitime", involving discretion in start and finish times, and the option to work from home. It is intended to improve working and social life for the employee by increasing time sovereignty.[1]

The relationship between work flexibility and health is an increasingly salient consideration due to changes in working norms induced by the coronavirus pandemic.[2–6] There is new impetus to understand the mechanisms via which any health benefits of flexible working occur, and for whom.

Beckers et al. provide a summary of the motivational and occupational health theories which give rise to the assumed benefits of increased worktime control.[7] Several are relevant for considering the effects that flexibility may have on employees' health. Effort-Recovery Theory [8] suggests that a flexible job may allow employees to improve their recovery after exerting effort at work, eg. by sleeping at times most optimal for them. Improved short-term recovery is likely to benefit physical and mental health in the long term.[9,10]

Through the lens of theories of work-home interaction,[11] flexibility allows employees to better meet obligations, needs, and activities in their private lives. This is particularly relevant for health, where many activities that employees undertake outside of work can directly affect health. An inflexible job may limit an individual's ability to engage in health promoting activities such as accessing healthcare, exercising, and cooking fresh food.[12]

Overall, the applied literature suggest that work flexibility may be positively associated with health and negatively associated with outcomes such as burnout, fatigue, perceived stress, and sickness absence[1,13–22]. A systematic review of the effect of flexibility on mental health concluded that flexibility had small positive effects on outcomes relating to mental health.[23]

However, previous studies suffer from several limitations. Most likely due to insufficient sample size, evidence of variation between population subgroups is lacking, even though we may expect some groups of employees to gain more from work flexibility (older employees and employees with other responsibilities).[24] Previous studies are also based predominantly

For peer review only - http://bmjopen.bmj.com/site/about/guidelines.xhtml

on work-focused surveys that tend to include only crude measures of health and no information on healthcare utilisation.

We were unable to find a study of the effect of work flexibility on health care utilisation, despite this being a potentially important pathway contributing to the overall effect of work flexibility on health. For employees working "standard" daytime hours, those are the same hours that most routine health care provision is available. Although there may be some provision outside of "standard" hours, employees may be more likely to forego health care appointments if their job does not afford them the flexibility to attend during working hours.

We use large, repeated, health-focused cross-sectional surveys for England to consider full-time employed individuals who report their jobs are so inflexible that they cannot attend primary care appointments during working hours. We map the geographic distribution of inflexible jobs, estimate the associations between job flexibility and health and healthcare utilisation, and exploit the large sample for subgroup analyses by gender, age, and deprivation.

METHODS

For peer review only

Study population

We used data from the national General Practice Patient Survey (GPPS) in England.[25] The survey is distributed by post to over two million people annually. It is sent to a sample of patients registered at each general practice in England and includes questions on job flexibility, health, primary healthcare use, socioeconomic characteristics, and the EQ-5D measure of health-related quality of life (HRQoL). We used data from six survey years, 2012 to 2017, when data on HRQoL was collected consistently using the five-level version of the EQ-5D (EQ-5D-5L).[26]

Procedures

If the respondent reported being in paid employment, they were asked: "*If you need to see a GP at your GP surgery during your typical working hours, can you take time away from your work to do this? Yes/No*".

For peer review only - http://bmjopen.bmj.com/site/about/guidelines.xhtml

The EQ-5D has five domains: ‘mobility’, ‘self-care’, ‘usual activities’, ‘pain/discomfort’, and ‘anxiety/depression’. Individuals were asked to report their health today in each domain on a five-point ordinal scale, where (1) corresponds to no problems; (2) slight problems; (3) moderate problems; (4) severe problems and (5) extreme problems/unable to do activities. We analysed each domain separately and a composite utility score, obtained by mapping the 5-level to the 3-level version using the cross-walk tool described in van Hout et al.[27]

We also analysed utilisation of three types of healthcare services. The first two types were GP and nurse visits. The GPPS asks how recently the respondent has seen or spoken to (1) a GP and (2) a nurse at their GP practice, with five possible responses ranging from *“in the past three months”* to *“I have never seen a [GP/nurse] from my surgery”*. We recoded these responses to create intervals for the number of months since the respondent had seen a GP/nurse (see Supplementary Table 1). The third type of healthcare was use of out-of-hours primary care. Up until 2015, the GPPS asked the question *“In the past 6 months, have you tried to call an out-of-hours GP service when [your usual] surgery was closed? Yes, for myself/ Yes, for someone else/ No”*. We considered only calls made for the respondent themself.

The GPPS contains information on the gender of each respondent, their age (in age bands: 18-24 years; 25-34 years; 35-44 years; 45-54 years; 55-64 years), their ethnicity (white; mixed; Asian; black; other), whether they had ever smoked, and whether they were a parent/guardian to a child aged 16 years and under, and whether they have a long-term condition.

The GPPS asks *“last time you wanted to see or speak to a nurse from your GP surgery, were you able to get an appointment to see or speak to someone?”*. We used the percentage of respondents who replied *“yes”* at each practice as an indicator of appointment availability at the practice level.

England is divided into 326 geographic areas called local authority districts (LADs), and is further broken down into a collection of 32,844 smaller geographic areas called lower-level super output areas (LSOAs). LSOAs contain on average 1,500 residents. Levels of deprivation were calculated at LSOA level using the 2015 iteration of the Index of Multiple Deprivation (IMD).[28] We used deciles of area deprivation, where 1 is the least deprived decile and 10 is the most deprived decile.

For peer review only - http://bmjopen.bmj.com/site/about/guidelines.xhtml

We restricted the main analysis to individuals who reported working full-time. We did this to avoid issues associated with part-time working. For example, we believe that part-time and full-time individuals face different choice sets when it comes to time allocation. As stated above, the main variable we are concerned with relates to an individual's ability to visit a GP during their *usual working hours*. This definition of usual working hours is likely to vary between full- and part-time employees. As a robustness check, we relaxed the assumption relating to full-time employment by including part-time workers in the estimation sample.

We also restricted the analysis to those within the statutory working-age range, 18-64 years. This is to minimise endogeneity generated by voluntary participation in work beyond state retirement age.

We retained observations with non-missing data for all outcomes and covariates. In the supplementary material we provide a flowchart which shows the number of observations dropped at each stage of data preparation (Supplementary Figure 1).

Statistical analyses

We calculated the percentage of GPPS respondents who reported having an inflexible job in each of the 326 LADs that existed as of 2017 in England. We used spmap [29] to create a heat map showing the percentage for each area. We compared this to a heat map of the mean EQ-5D utility score for GPPS respondents in each area.

We used linear regression to estimate the strength of the overall association between the EQ-5D composite utility score and having an inflexible job, adjusting for person characteristics, LSOA characteristics, and fixed effects for local authorities and survey years. We used ordered logistic regression to analyse the EQ-5D domains.

We used interval regression[30] to estimate the strength of association between the number of months since the respondent last saw (1) a GP or (2) a nurse and having an inflexible job; and logistic regression for whether the respondent had tried to access out-of-hours primary care in the past six months. We included the same covariates in these regressions in addition to: indicators for specific long-term conditions, and an indicator of appointment availability at the practice level.

The main analyses do not combine the associations of job inflexibility with health and with healthcare utilisation. As shown in Figure 1, it is possible that healthcare utilisation is one of

For peer review only - http://bmjopen.bmj.com/site/about/guidelines.xhtml

the reasons health is adversely affected. If having an inflexible job acts as a barrier to accessing healthcare when it is needed, this would result in a negative effect on health care use. Because health care is expected to have a positive effect on health, this would create an additional negative effect of an inflexible job on health.

To test this pathway, we used two-stage least squares (2SLS) to decompose the effect of health care utilisation on health into that which is related to (1) having an inflexible job, and (2) all other factors that determine health care utilisation. In the first stage, we estimated the association between the time since the respondent last saw a GP or nurse, and whether they have an inflexible job. In the second stage, we estimated the association between the predicted values for the time since the respondent last saw a GP/nurse and HRQoL. In these regressions, we treat the time since the respondent last saw a GP/nurse as a continuous variable ranging from one to five (Supplementary Table 1).

We present the equations for each regression specification as supplementary material.

We estimated all models with robust standard errors, and used the weights provided with the survey data which correct for the GPPS sampling design and reduce the impact of non-response bias.

To investigate differences between subgroups, we estimated all models for men and women separately. We additionally estimated the effect of having an inflexible job on HRQoL with interactions between having an inflexible job and: gender, area deprivation decile, and age group.

Patient and public involvement

Patients or the public were not involved in the design of this study.

RESULTS

The estimation sample comprised 570,626 women and 662,258 men, aged 18-64 years and in full-time employment. Thirty-two percent of respondents reported that their job was inflexible. There were gender differences, with men reporting lower levels than women (29% compared to 35%; Table 1).

For peer review only - http://bmjopen.bmj.com/site/about/guidelines.xhtml

Panel (a) of Supplementary Figure 2 shows the geographic distribution of inflexible jobs. The areas with the highest concentration of inflexible jobs (darkest shading) were areas of the north-east, the north-west, west Midlands, and London. These areas are traditionally the areas with the highest levels of deprivation and the highest concentration of manual jobs. Supplementary Tables 2 and 3 provide greater detail on which local authorities had the highest and lowest percentages of respondents who reported inflexible jobs.

Panel (b) of Supplementary Figure 2 shows the geographic distribution of mean EQ-5D utility score. Darker areas indicate worse health, and these are present in both the north and the south. London has a high concentration of light-coloured areas which represent good average health. Excluding London, there was an apparent correlation between the two measures: areas with higher rates of inflexible jobs, on average, had lower levels of health. The raw individual level correlation of -0.051 between reporting an inflexible job and EQ-5D utility score confirms this.

The mean EQ-5D utility score was 0.89, for both women and men. The domain with the highest percentage of respondents reporting "no problems" was self-care (98%; Supplementary Table 4). In descending order, the percentage of respondents reporting "no problems" in other domains were: mobility (91%); usual activities (88%); anxiety/depression (74%); and pain/discomfort (64%). All domains were very similar across gender, except anxiety/depression, where 77% of men reported "no problems" compared with 71% of women.

41% of respondents reported that they have a long-term condition. The most prevalent long-term condition among males was high blood pressure (10%); "another long-term condition" was the most prevalent for females (10%; Supplementary Table 5).

35% of men and 25% of women were parents, and the modal age group was 45-54 years of age. The sample was predominantly white (88%), with 7% Asian, 3% black, 2% other and 1% mixed (Table 1).

Individuals in the sample tended to have seen a GP more recently than a nurse (Table 1). 44% saw a GP in the last six months, compared to 26% who saw a nurse. 2% of individuals had never seen a GP in their practice, compared to 10% who had never seen a nurse.

For peer review only - http://bmjopen.bmj.com/site/about/guidelines.xhtml

Table 1: Selected summary statistics for males and females

	Female	Male
Outcomes		
Mean EQ-5D utility score [SD]	0.89 [0.14]	0.89 [0.14]
Months since last saw GP (%)		
0 to 3	53.8	38.4
3 to 6	19.2	18.2
6 to 12	14.6	18.0
12 to 24	11.3	22.8
24+	1.1	2.7
Months since last saw nurse (%)		
0 to 3	33.8	21.1
3 to 6	18.9	13.4
6 to 12	19.1	15.7
12 to 24	24.0	35.9
24+	4.2	13.8
Out-of-hours (% contacted in last 6 months)	3.2	2.2
Key variable (%)		
Inflexible job	35.4	28.9
Individual level characteristics (%)		
Parent	24.7	34.9
Aged 18-24 years	11.0	7.6
Aged 25-34 years	28.5	23.9
Aged 35-44 years	22.6	25.9
Aged 45-54 years	25.9	27.1
Aged 55-64 years	12.1	15.5
Ethnicity: white	88.8	86.7
Ethnicity: mixed	1.3	1.0
Ethnicity: Asian	5.5	7.5
Ethnicity: black	2.8	2.4
Ethnicity: other	1.6	2.3
Never smoked	62.7	56.4
General practice level covariates		
Mean appointment availability [SD]	90.2 [6.0]	90.2 [6.1]
	N=570,626	N=662,258

Data source: GPPS pooled data for 2012 to 2017; respondents in full-time employment.
Weighted using GPPS weights.

Respondents living in more deprived areas were more likely to have an inflexible job compared to those living in less deprived areas (Table 2; columns (1) and (2)). Older respondents were less likely than younger respondents to report having an inflexible job. Women were more likely to have an inflexible job than men (Supplementary Table 6; column (1)).

For peer review only - http://bmjopen.bmj.com/site/about/guidelines.xhtml

Women whose jobs were inflexible had average EQ-5D utility scores that were 0.017 (95% confidence interval (CI) [0.016; 0.018]) lower than their counterparts with a flexible job (Table 2; column (3)). Men whose jobs were inflexible had average EQ-5D utility scores that were 0.016 (95% CI [0.015; 0.017]) lower than their counterparts with a flexible job (Table 2; column (4)). The standard deviation of the EQ-5D utility score was 0.14 for men and women (Table 1). This means that the reduction was about 12% of one standard deviation for women and 11% for men.

These effects remain when part time workers are included in the estimation sample (Supplementary Table 7). The main coefficients of interest (on 'inflexible job') are almost identical in magnitude and statistical significance.

For peer review only

Table 2: The effect of covariates on reporting an inflexible job and the effect of an inflexible job on the EQ-5D utility score

	**Inflexible job**		**EQ-5D utility score**	
	Females (1)	Males (2)	Females (3)	Males (4)
Inflexible job			-0.017	-0.016
			[-0.018; -0.016]	[-0.017; -0.015]
Parent	-0.005	0.002	0.018	0.011
	[-0.008; -0.001]	[-0.002; 0.005]	[0.017; 0.019]	[0.010; 0.012]
Aged 25-34 years [a]	-0.064	-0.061	0.001	-0.019
	[-0.071; -0.057]	[-0.070; -0.053]	[-0.001; 0.003]	[-0.021; -0.017]
Aged 35-44 years [a]	-0.097	-0.089	-0.019	-0.038
	[-0.104; -0.090]	[-0.098; -0.081]	[-0.021; -0.017]	[-0.040; -0.036]
Aged 45-54 years [a]	-0.106	-0.101	-0.038	-0.056
	[-0.113; -0.099]	[-0.109; -0.093]	[-0.040; -0.036]	[-0.058; -0.054]
Aged 55-64 years [a]	-0.134	-0.131	-0.050	-0.071
	[-0.141; -0.127]	[-0.139; -0.124]	[-0.052; -0.048]	[-0.073; -0.069]
Ethnicity: mixed [b]	0.002	0.001	-0.010	-0.004
	[-0.012; 0.016]	[-0.014; 0.017]	[-0.014; -0.005]	[-0.009; 0.000]
Ethnicity: Asian [b]	0.006	0.022	0.005	0.008
	[-0.001; 0.013]	[0.016; 0.028]	[0.003; 0.007]	[0.006; 0.009]
Ethnicity: black [b]	-0.034	-0.011	0.001	0.017
	[-0.043; -0.025]	[-0.020; -0.002]	[-0.002; 0.003]	[0.015; 0.020]
Ethnicity: other [b]	-0.000	0.013	-0.010	-0.004
	[-0.012; 0.012]	[0.003; 0.023]	[-0.014; -0.006]	[-0.008; -0.001]
Never smoked	0.012	-0.024	0.026	0.025
	[0.009; 0.016]	[-0.027; -0.021]	[0.025; 0.027]	[0.024; 0.026]
Deprivation decile 1 [c]	-0.007	-0.031	0.009	0.011

(least deprived)	[-0.014; 0.000]	[-0.038; -0.025]	[0.007; 0.011]	[0.009; 0.013]
Deprivation decile 2 [c]	-0.012	-0.019	0.006	0.008
	[-0.019; -0.005]	[-0.026; -0.013]	[0.004; 0.008]	[0.006; 0.010]
Deprivation decile 3 [c]	-0.010	-0.018	0.004	0.006
	[-0.017; -0.003]	[-0.024; -0.011]	[0.002; 0.006]	[0.004; 0.007]
Deprivation decile 4 [c]	-0.005	-0.004	0.002	0.003
	[-0.012; 0.002]	[-0.011; 0.002]	[-0.000; 0.003]	[0.001; 0.005]
Deprivation decile 6 [c]	0.004	0.006	-0.005	-0.003
	[-0.003; 0.011]	[-0.000; 0.013]	[-0.007; -0.003]	[-0.005; -0.001]
Deprivation decile 7 [c]	0.010	0.016	-0.010	-0.008
	[0.003; 0.017]	[0.010; 0.023]	[-0.012; -0.008]	[-0.010; -0.006]
Deprivation decile 8 [c]	0.015	0.024	-0.012	-0.010
	[0.008; 0.022]	[0.018; 0.031]	[-0.014; -0.010]	[-0.012; -0.008]
Deprivation decile 9 [c]	0.029	0.041	-0.016	-0.013
	[0.022; 0.036]	[0.034; 0.048]	[-0.018; -0.014]	[-0.015; -0.011]
Deprivation decile 10 [c]	0.042	0.053	-0.023	-0.018
(most deprived)	[0.035; 0.050]	[0.046; 0.061]	[-0.025; -0.021]	[-0.020; -0.015]
N	570,626	662,258	570,626	662,258
Adjusted R^2	0.013	0.018	0.040	0.044

Data source: GPPS pooled data for 2012 to 2017.
95% confidence intervals in parentheses.
[a] The reference category for age group is 16-24 years.
[b] The reference category for ethnic group is white.
[c] The reference category for deprivation is decile 5.
Models estimated using OLS.
Models additionally include fixed effects for survey year and Local Authority District of residence.

For the five separate domains of the EQ-5D, having an inflexible job was associated with a lower probability of reporting "no problems", and a greater probability of all other responses (Table 3). The magnitude of the effects on the probability of reporting "no problems" were slightly larger in absolute magnitude for men in the domains of mobility, self-care, usual activities, and pain/discomfort. However, for the anxiety/depression domain, the effect was larger for women than men.

There was both a deprivation and age gradient on the effect of an inflexible job on health (Figure 2; Supplementary Table 6, column (2)). The interaction effects suggest that the negative effect of having an inflexible job on health was larger in magnitude for people living in a more deprived area compared to those living in a less deprived area; larger in magnitude for older people compared to younger people; and very slightly larger for women, compared with men.

For peer review only

Having an inflexible job was associated with less recent utilisation of both GPs and nurses (Table 4; Supplementary Table 8). For women with an inflexible job, it had been an additional 0.234 (95% CI [0.201; 0.268]) months since they had seen a GP at their surgery, compared to women with a flexible job. The magnitude of this effect was greater for nurse appointments than GP appointments; for women with an inflexible job it had been an additional 0.569 (95% CI [0.519; 0.618]) months since they had seen a nurse at their GP surgery, compared to women with a flexible job. For men with an inflexible job, it had been an additional 0.199 (95% CI [0.152; 0.247]) months since they last saw a GP at their surgery, and an additional 0.653 (95% CI [0.586; 0.720]) months since they last saw a nurse at their GP surgery, compared to men with a flexible job. These results are robust to a range of alternative definitions of each interval in the interval regressions (Supplementary Tables 9 and 10).

Conversely, individuals with inflexible jobs made more use of 'out-of-hours' GP appointments than their counterparts with flexible jobs (Table 4; Supplementary Table 11).

The results of the second stage of the two-stage least squares regression show the effect of health care use on the EQ-5D utility score when healthcare use is predicted by job inflexibility (Table 5; Supplementary Table 12). A longer predicted duration of time since the individual had seen a GP (or nurse) was negatively associated with the EQ-5D utility score. Therefore, the element of healthcare use that is negatively associated with job inflexibility is also associated with lower health.

For peer review only

For peer review only - http://bmjopen.bmj.com/site/about/guidelines.xhtml

Table 3: The average marginal effect of having an inflexible job on the probability of reporting each EQ-5D domain level

	Mobility		Self-care		Usual activities		Pain/discomfort		Anxiety/depression	
	Females	Males	Females	Males	Females	Males	Females	Males	Females	Males
No problems	-0.014	-0.017	-0.002	-0.003	-0.023	-0.025	-0.051	-0.052	-0.035	-0.021
	[-0.015; -0.012]	[-0.019; -0.015]	[-0.003; -0.002]	[-0.004; -0.002]	[-0.026; -0.021]	[-0.027; -0.023]	[-0.054; -0.048]	[-0.055; -0.049]	[-0.038; -0.032]	[-0.024; -0.018]
Slight problems	0.010	0.012	0.002	0.002	0.017	0.018	0.030	0.033	0.020	0.014
	[0.008; 0.011]	[0.011; 0.013]	[0.001; 0.002]	[0.002; 0.003]	[0.016; 0.019]	[0.016; 0.019]	[0.028; 0.032]	[0.031; 0.035]	[0.019; 0.023]	[0.012; 0.015]
Moderate problems	0.003	0.004	0.001	0.001	0.005	0.005	0.017	0.016	0.011	0.006
	[0.003; 0.003]	[0.003; 0.004]	[0.0004; 0.0007]	[0.0005; 0.0008]	[0.005; 0.006]	[0.005; 0.006]	[0.016; 0.018]	[0.015; 0.017]	[0.010; 0.012]	[0.005; 0.007]
Severe problems	0.001	0.001	0.0001	0.0001	0.001	0.001	0.004	0.003	0.002	0.001
	[0.001; 0.001]	[0.001; 0.001]	[0.0001; 0.0001]	[0.0001; 0.0001]	[0.001; 0.001]	[0.001; 0.001]	[0.004; 0.004]	[0.003; 0.003]	[0.002; 0.002]	[0.001; 0.001]
Unable to / extreme problems	0.0002	0.0003	0.0001	0.0001	0.0004	0.0004	0.001	0.0005	0.001	0.0003
	[0.0002;0.0002]	[0.0002;0.0003]	[0.0001; 0.0001]	[0.0001; 0.0002]	[0.0004; 0.0005]	[0.004; 0.0005]	[0.001; 0.001]	[0.0004; 0.0005]	[0.0005; 0.001]	[0.0003; 0.0004]
N	570,626	662,258	570,626	662,258	570,626	662,258	570,626	662,258	570,626	662,258
Pseudo R^2	0.044	0.045	0.033	0.033	0.023	0.029	0.028	0.031	0.012	0.011

Data source: GPPS pooled data for 2012 to 2017.
95% confidence intervals in parentheses.
Models additionally include indicators for: parental status, ten-year age group, ethnicity, whether the respondent has ever smoked, index of multiple deprivation decile and fixed effects for survey year and Local Authority District of residence.
Models estimated using ordered logistic regression.

For peer review only

Table 4: The effects of an inflexible job on health care utilisation

	Months since last saw GP		Months since last saw nurse		Tried to call out-of-hours service in past 6 months	
	Females (1)	Males (2)	Females (3)	Males (4)	Females (5)	Males (6)
Inflexible job	0.234 [0.201; 0.268]	0.199 [0.152; 0.247]	0.569 [0.519; 0.618]	0.653 [0.586; 0.720]	0.012 [0.010; 0.015]	0.010 [0.008; 0.012]
N	570,626	662,258	570,626	662,258	293,266	336,890
Pseudo R^2					0.028	0.260
AIC	2,203,151	3,576,803	2,317,874	3,453,027		
BIC	2,207,315	3,581,022	2,322,038	3,457,246		

Data source (2) to (4): GPPS pooled data for 2012 to 2017.
Data source (5) and (4): GPPS pooled data for 2012 to 2014.
95% confidence intervals in parentheses.
All models additionally include indicators for: parental status, ten-year age group, ethnicity, whether the respondent has ever smoked, index of multiple deprivation decile, 16 long-term conditions, practice level appointment availability, and fixed effects for survey year and Local Authority District of residence.
Models (1) to (4) estimated using interval regression.
Models (5) and (6) estimated using logistic regression, results show average marginal effects.

For peer review only

Table 5: Two-stage least squares regression results: the mediating effect of GP visits in the effect of an inflexible job on health

	**First stage: Time since last saw GP**		**Second stage: EQ-5D utility score**	
	Females (1)	Males (2)	Females (3)	Males (4)
Time since last saw GP			-0.355	-0.539
			[-0.419; -0.291]	[-0.715; -0.362]
Inflexible job	0.047	0.029		
	[0.039; 0.054]	[0.020; 0.038]		
Parent	-0.279	-0.140	-0.082	-0.064
	[-0.288; -0.271]	[-0.149; -0.130]	[-0.100; -0.063]	[-0.090; -0.039]
Aged 25-34 years [a]	0.025	-0.060	0.01	-0.051
	[0.010; 0.041]	[-0.082; -0.038]	[0.004; 0.016]	[-0.068; -0.035]
Aged 35-44 years [a]	0.14	-0.169	0.03	-0.129
	[0.124; 0.156]	[-0.190; -0.147]	[0.020; 0.041]	[-0.161; -0.096]
Aged 45-54 years [a]	0.161	-0.279	0.019	-0.206
	[0.146; 0.175]	[-0.300; -0.259]	[0.007; 0.031]	[-0.258; -0.155]
Aged 55-64 years [a]	0.146	-0.486	0.002	-0.333
	[0.130; 0.161]	[-0.507; -0.466]	[-0.009; 0.013]	[-0.420; -0.246]
Ethnicity: mixed [b]	-0.029	-0.064	-0.02	-0.039
	[-0.060; 0.002]	[-0.106; -0.022]	[-0.033; -0.007]	[-0.065; -0.012]
Ethnicity: Asian [b]	0.014	-0.227	0.01	-0.115
	[-0.002; 0.031]	[-0.243; -0.211]	[0.003; 0.016]	[-0.156; -0.074]
Ethnicity: black [b]	-0.11	-0.182	-0.038	-0.080
	[-0.129; -0.091]	[-0.205; -0.158]	[-0.049; -0.028]	[-0.115; -0.046]
Ethnicity: other [b]	-0.022	-0.185	-0.018	-0.104
	[-0.051; 0.007]	[-0.212; -0.159]	[-0.029; -0.006]	[-0.140; -0.068]
Never smoked	0.114	0.101	0.067	0.080
	[0.107; 0.121]	[0.093; 0.109]	[0.059; 0.075]	[0.061; 0.098]
Deprivation decile 1 [c]	0.007	0.027	0.012	0.025
(least deprived)	[-0.010; 0.023]	[0.008; 0.045]	[0.005; 0.018]	[0.014; 0.037]
Deprivation decile 2 [c]	-0.003	0.016	0.005	0.016
	[-0.019; 0.013]	[-0.002; 0.034]	[-0.002; 0.011]	[0.006; 0.027]
Deprivation decile 3 [c]	0.008	-0.000	0.007	0.005
	[-0.008; 0.024]	[-0.018; 0.018]	[0.001; 0.013]	[-0.005; 0.016]
Deprivation decile 4 [c]	-0.012	0.000	-0.003	0.003
	[-0.028; 0.003]	[-0.018; 0.018]	[-0.009; 0.003]	[-0.007; 0.014]
Deprivation decile 6 [c]	0.002	-0.008	-0.004	-0.008
	[-0.014; 0.018]	[-0.026; 0.010]	[-0.010; 0.002]	[-0.018; 0.003]
Deprivation decile 7 [c]	-0.028	-0.011	-0.02	-0.014
	[-0.044; -0.013]	[-0.030; 0.007]	[-0.026; -0.013]	[-0.025; -0.003]
Deprivation decile 8 [c]	-0.018	-0.007	-0.019	-0.013
	[-0.034; -0.002]	[-0.025; 0.012]	[-0.025; -0.012]	[-0.024; -0.003]
Deprivation decile 9 [c]	-0.014	-0.022	-0.021	-0.025

For peer review only

	[-0.031; 0.002]	[-0.041; -0.003]	[-0.027; -0.014]	[-0.036; -0.013]
Deprivation decile 10 [c] (most deprived)	-0.03 [-0.047; -0.013]	-0.037 [-0.056; -0.017]	-0.033 [-0.041; -0.026]	-0.037 [-0.050; -0.025]
N	570,626	662,258	570,626	662,258

Data source: GPPS pooled data for 2012 to 2017.
95% confidence intervals in parentheses.
[a] The reference category for age group is 16-24 years.
[b] The reference category for ethnic group is white.
[c] The reference category for deprivation is decile 5.
Models additionally include fixed effects for survey year and Local Authority District of residence.

DISCUSSION

We found that individuals who had inflexible jobs were less likely to be in good health and the result was common to men and women. Our subgroup analyses of different age groups and people living in areas of differing deprivation suggest that the most affected groups were older aged people and those living in the most deprived areas. Older aged people were less likely to experience inflexible jobs, but their health was more affected if they did, compared to younger people. People living in more deprived areas were both more likely to have an inflexible job, and their health was more affected than those in less deprived areas.

When considering subdomains of health separately, we found that women were more affected in the anxiety/depression domain than men. This is a similar finding to Moen et al.[13] which found that women especially benefitted from a reduction in perceived stress and psychological stress when randomised into an intervention with greater work-time control.

We found that individuals with inflexible jobs made fewer visits to see their GP or a nurse compared to individuals with flexible jobs. As would perhaps be expected, individuals with inflexible jobs used out-of-hours services more often than individuals with flexible jobs, but still overall went to see their GP/nurse less frequently. It is possible that the lower opportunity cost of time for workers who were able to consume healthcare during working hours contributed to their more frequent visits to their GP.

Typically, the onus has been put on the health care system to accommodate inflexible jobs by offering “out-of-hours” appointments in the evenings and on weekends, at greater cost to the NHS than regular GP appointments. However, perhaps there should be a greater responsibility for employers to ensure that systems are in place so that their employees are

For peer review only - http://bmjopen.bmj.com/site/about/guidelines.xhtml

able to attend routine health care appointments during usual working hours, when they need to.

We add to the evidence base on relationship between work flexibility and health, and to the very limited evidence base on the relationship between work flexibility and health care utilisation. Our analyses suggest that having an inflexible job represents an additional barrier to utilising routine health care, which may be one factor contributing to negative health effects of inflexible jobs.

The occupations which have seen increased home working during the pandemic (typically those where work can be carried out using IT workarounds) are disproportionately those which are well paid [31] and require higher qualifications and more experience.[2] An increase in flexible working in these occupations could make already "good" jobs even better, widening the gap between "good" jobs and "bad" jobs.[32] This could contribute to widening inequalities in health.

Limitations

There are well-known empirical barriers to establishing a causal relationship between job characteristics and health. In particular, people with certain unobservable characteristics have a tendency to self-select into certain occupations. Part of the association we found between inflexible jobs and health is likely to be due to self-selection and/or reverse causality.

The people we identified as having inflexible jobs may also have been more likely to have jobs with other "bad" characteristics such as a highly stressful work environment or a casual employment contract. We were unable to control for other job characteristics at an individual level, as this information is not included in the GPPS. Therefore, it is possible that the estimated effect of job inflexibility may be partly attributable to a correlated job characteristic. Data on employment composition are available at the LAD level, however when we included indicators for the percentage share of each occupational classification in the regression models they were jointly insignificant.

We identified people who had an inflexible job as those who responded that they were unable to go to see their GP in their usual working hours. There are, in fact, two possible types of job that would result in giving this response: those who worked "normal hours" (eg. 9am to 5pm) but were unable to take time off during those hours to visit their GP; and those who worked unsociable hours, so could not visit their GP during their working hours because the GP

For peer review only - http://bmjopen.bmj.com/site/about/guidelines.xhtml

service was not open. Both of these job features are less desirable and may have a harmful effect on health.

The source of individual level data we used is from a survey intended to gather information on patients' experience of general practice (GPPS). It is possible that people were more likely to respond to the survey if they had used their general practice recently. We used the survey weights provided with the GPPS to adjust for this, but it is still possible that this may affect generalisability to the general population.

Conclusion

This is the first study to examine the relationship between job flexibility and health and health care utilisation in a large dataset. We found that having an inflexible job – defined as not being able to take time away from work during usual working hours to visit a medical professional – was associated with reduced HRQoL and less recent visits to see a GP or nurse. This relationship was consistent for both men and women.

Individuals living in more deprived areas were more likely to report having an inflexible job, and this inflexible job was more likely to have a negative impact on their health. This suggests that flexible working may have the potential to either exacerbate or reduce existing inequalities in health, depending on how it is distributed throughout the working population.

DECLARATIONS

Ethics approval and consent to participate

Not applicable

Consent for publication

Not applicable

Availability of data and materials

The General Practice Patient Survey data that support the findings of this study are available from NHS England but restrictions apply to the availability of these data, which were used under license for the current study, and so are not publicly available.

For peer review only - http://bmjopen.bmj.com/site/about/guidelines.xhtml

For peer review only

Competing interests

The authors declare that they have no competing interests.

Funding

This research was supported by the National Institute for Health Research Applied Research Collaboration Greater Manchester [NIHR200174]. Charlie Moss received funding from a National Institute for Health Research (NIHR) Research Methods Fellowship [RM-FI-2017-08-018]. Luke Munford was supported by the Medical Research Council, through a Skills Development Fellowship [MR/N015126/1]. Matt Sutton is an NIHR Senior Investigator. The views expressed are those of the authors and not necessarily those of the MRC, the NIHR or the Department of Health and Social Care.

Authors' contributions

MS had the original idea for the study. MS, LM and CM designed the analysis. CM carried out the analysis. CM, LM and MS contributed substantially to the interpretation of results and the writing of the manuscript.

Acknowledgements

We are grateful for comments and suggestions received from colleagues at HOPE and the Melbourne Institute: Applied Economic and Social Research. Additional thanks to Nordic Health Economists' Study Group 2019 conference attendees for a useful discussion of a previous version.

REFERENCES

1 Costa PG, Sartori S, Åkerstedt T. Influence of Flexibility and Variability of Working Hours on Health and Well-Being. *Chronobiology International* 2006;**23**:1125–37. doi:10.1080/07420520601087491

2 Office for National Statistics. Coronavirus and homeworking in the UK. 2020.https://www.ons.gov.uk/employmentandlabourmarket/peopleinwork/employmentandemployeetypes/bulletins/coronavirusandhomeworkingintheuk/april2020 (accessed 5 Nov 2020).

3 Bloom N. How working from home works out. Stanford Institute for Economic Policy Research 2020.

For peer review only - http://bmjopen.bmj.com/site/about/guidelines.xhtml

https://siepr.stanford.edu/sites/default/files/publications/SIEPR%20Policy%20Brief%20June%202020%20v04.pdf (accessed 11 May 2020).

4 Peters SE, Dennerlein JT, Wagner GR, *et al.* Work and worker health in the post-pandemic world: a public health perspective. *The Lancet Public Health* 2022;**7**:e188–94. doi:10.1016/S2468-2667(21)00259-0

5 The Economist. Covid-19 has forced a radical shift in working habits. *The Economist* 2020.https://www.economist.com/briefing/2020/09/12/covid-19-has-forced-a-radical-shift-in-working-habits (accessed 5 Nov 2020).

6 Kramer A, Kramer KZ. The potential impact of the Covid-19 pandemic on occupational status, work from home, and occupational mobility. *Journal of Vocational Behavior* 2020;**119**:103442. doi:10.1016/j.jvb.2020.103442

7 Beckers DG, Kompier MA, Kecklund G, *et al.* Worktime control: theoretical conceptualization, current empirical knowledge, and research agenda. *Scandinavian Journal of Work, Environment & Health* 2012;**38**:291–7.

8 Meijman TF, Mulder G. Psychological Aspects of Workload. In: *A Handbook of Work and Organizational Psychology*. Psychology Press 1998.

9 Sluiter JK, Croon EM de, Meijman TF, *et al.* Need for recovery from work related fatigue and its role in the development and prediction of subjective health complaints. *Occupational and Environmental Medicine* 2003;**60**:i62–70. doi:10.1136/oem.60.suppl_1.i62

10 von Thiele U, Lindfors P, Lundberg U. Self-rated recovery from work stress and allostatic load in women. *Journal of Psychosomatic Research* 2006;**61**:237–42. doi:10.1016/j.jpsychores.2006.01.015

11 Geurts SAE, Demerouti E. Work/Non-Work Interface: A Review of Theories and Findings. In: *The Handbook of Work and Health Psychology*. John Wiley & Sons, Ltd 2002. 279–312. doi:10.1002/0470013400.ch14

12 Restrepo BJ, Zeballos E. The effect of working from home on major time allocations with a focus on food-related activities. *Rev Econ Household* 2020;**18**:1165–87. doi:10.1007/s11150-020-09497-9

13 Moen P, Kelly EL, Fan W, *et al.* Does a Flexibility/Support Organizational Initiative Improve High-Tech Employees' Well-Being? Evidence from the Work, Family, and Health Network. *Am Sociol Rev* 2016;**81**:134–64. doi:10.1177/0003122415622391

14 Lee S, Almeida DM, Berkman L, *et al.* Age differences in workplace intervention effects on employees' nighttime and daytime sleep. *Sleep Health* 2016;**2**:289–96. doi:10.1016/j.sleh.2016.08.004

15 Takahashi M, Iwasaki K, Sasaki T, *et al.* Sleep, Fatigue, Recovery, and Depression After Change in Work Time Control: A One-year Follow-Up Study. *Journal of Occupational and Environmental Medicine* 2012;**54**:1078–85. doi:10.1097/JOM.0b013e31826230b7

For peer review only

For peer review only - http://bmjopen.bmj.com/site/about/guidelines.xhtml

16 Almeida DM, Lee S, Walter KN, *et al.* The effects of a workplace intervention on employees' cortisol awakening response. *Community, Work & Family* 2018;**21**:151–67. doi:10.1080/13668803.2018.1428172

17 Joyce K, Pabayo R, Critchley JA, *et al.* Flexible working conditions and their effects on employee health and wellbeing. *Cochrane Database of Systematic Reviews* Published Online First: 2010. doi:10.1002/14651858.CD008009.pub2

18 Nijp HH, Beckers DG, Geurts SA, *et al.* Systematic review on the association between employee worktime control and work-non-work balance, health and well-being, and job-related outcomes. *Scandinavian Journal of Work, Environment & Health* 2012;**38**:299–313.

19 Brauner C, Wöhrmann AM, Frank K, *et al.* Health and work-life balance across types of work schedules: A latent class analysis. *Applied Ergonomics* 2019;**81**:102906. doi:10.1016/j.apergo.2019.102906

20 Albrecht SC, Kecklund G, Rajaleid K, *et al.* The longitudinal relationship between control over working hours and depressive symptoms: Results from SLOSH, a population-based cohort study. *Journal of Affective Disorders* 2017;**215**:143–51. doi:10.1016/j.jad.2017.03.010

21 Ropponen A, Känsälä M, Rantanen J, *et al.* Organizational Initiatives for Promoting Employee Work-Life Reconciliation Over the Life Course. A Systematic Review of Intervention Studies. *Nordic Journal of Working Life Studies* 2016;**6**. doi:10.19154/njwls.v6i3.5529

22 Nätti J, Oinas T, Anttila T. Time pressure, working time control and long-term sickness absence. *Occup Environ Med* 2015;**72**:265–70. doi:10.1136/oemed-2014-102435

23 Shiri R, Turunen J, Kausto J, *et al.* The Effect of Employee-Oriented Flexible Work on Mental Health: A Systematic Review. *Healthcare* 2022;**10**:883. doi:10.3390/healthcare10050883

24 Office for National Statistics. Living longer: impact of working from home on older workers. 2021. https://www.ons.gov.uk/peoplepopulationandcommunity/birthsdeathsandmarriages/ageing/articles/livinglongerimpactofworkingfromhomeonolderworkers/2021-08-25 (accessed 4 Nov 2021).

25 GP Patient Survey. About the survey. 2020.https://www.gp-patient.co.uk/About (accessed 19 Aug 2020).

26 Brooks R. EuroQol: the current state of play. *Health Policy* 1996;**37**:53–72. doi:10.1016/0168-8510(96)00822-6

27 van Hout B, Janssen MF, Feng Y-S, *et al.* Interim Scoring for the EQ-5D-5L: Mapping the EQ-5D-5L to EQ-5D-3L Value Sets. *Value in Health* 2012;**15**:708–15. doi:10.1016/j.jval.2012.02.008

For peer review only - http://bmjopen.bmj.com/site/about/guidelines.xhtml

28 Office for National Statistics. English indices of deprivation 2015. GOV.UK. 2015.https://www.gov.uk/government/statistics/english-indices-of-deprivation-2015 (accessed 4 Nov 2020).

29 Pisati M. *SPMAP: Stata module to visualize spatial data*. 2018. https://econpapers.repec.org/software/bocbocode/s456812.htm (accessed 23 Aug 2020).

30 Stewart MB. On Least Squares Estimation when the Dependent Variable is Grouped. *The Review of Economic Studies* 1983;**50**:737–53. doi:10.2307/2297773

31 Dingel JI, Neiman B. How Many Jobs Can be Done at Home? National Bureau of Economic Research 2020. doi:10.3386/w26948

32 Wu N. Flexible Working: Are We Ready for This? In: Caven V, Nachmias S, eds. *Hidden Inequalities in the Workplace: A Guide to the Current Challenges, Issues and Business Solutions*. Cham: : Springer International Publishing 2018. 127–54. doi:10.1007/978-3-319-59686-0_6

For peer review only

For peer review only - http://bmjopen.bmj.com/site/about/guidelines.xhtml

Figure legends

Figure 1: Graph of the hypothesised effect of an inflexible job on health

Figure 2: Subgroup effects: The interaction between having an inflexible job and deprivation decile (left) and age (right)

Notes:

Data source: GPPS pooled data for 2012 to 2017; 1,232,884 respondents in full-time employment.

Markers show point estimates of interaction terms; bars show 95% confidence intervals.

Model estimated using OLS.

Model additionally includes indicators for: gender, gender*inflexible job, parental status, ten-year age group, ethnicity, whether the respondent has ever smoked, index of multiple deprivation decile, and fixed effects for survey year and Local Authority District of residence.

For peer review only - http://bmjopen.bmj.com/site/about/guidelines.xhtml

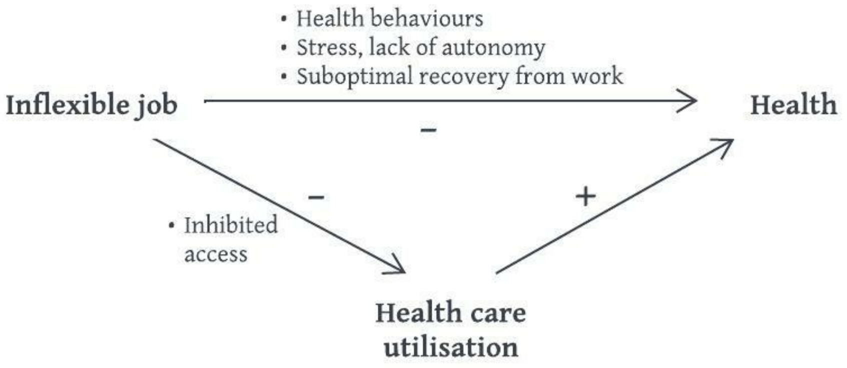

Figure 1: Graph of the hypothesised effect of an inflexible job on health

141x63mm (160 x 160 DPI)

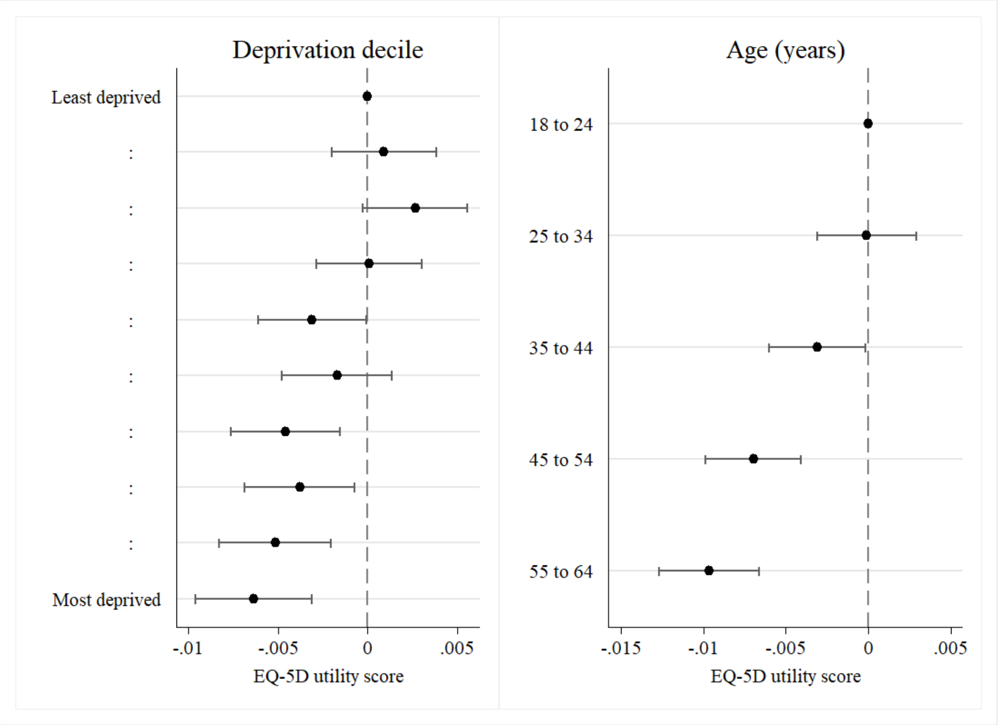

Figure 2: Subgroup effects: The interaction between having an inflexible job and deprivation decile (left) and age (right)

388x282mm (72 x 72 DPI)

SUPPLEMENTARY MATERIAL

Regression equations

1. The effect of having an inflexible job on EQ-5D utility score and EQ-5D domains:

$$H_{ijl} = \beta . inflexible_{ijl} + \boldsymbol{\gamma_1 X_{ij}} + \tau_l + \delta_t + \varepsilon_{ijl} \quad (1)$$

Where *H* are the health outcomes of individual *i* living in LSOA *j* in local authority *l*;

inflexible is an indicator equal to one if an individual reports that their job is inflexible;

X_{ij} is a vector of covariates at individual and LSOA level including indicators for gender, ten-year age band, index of multiple deprivation decile, parental status, ethnicity, and whether the respondent ever smoked;

τ_l are LAD fixed-effects;

δ_t are time fixed-effects, and ε_{ijl} is an error term.

2. The effect of having an inflexible job on the length of time since the respondent last saw a GP or nurse, and use of out-of-hours (healthcare utilisation):

$$HCU_{ijlp} = \beta . inflexible_{ijl} + \boldsymbol{\gamma_1 X_{ij}} + \boldsymbol{\gamma_2 LTC_i} + \gamma_3 . availability_p + \tau_l + \delta_t + \varepsilon_{ijl} \quad (2)$$

Where *HCU* are the healthcare utilisation outcomes of individual *i* living in LSOA *j* in local authority *l* registered at general practice *p*;

LTC_i is a vector of indicators for whether individual *i* has each long-term condition;

$availability_p$ is a measure of the average appointment availability at practice *p*;

inflexible, X_{ij}, τ_l, δ_t and ε_{ijl} are as in equation (1).

3. The mediating effect of health care use in the relationship between having an inflexible job and health:

For peer review only - http://bmjopen.bmj.com/site/about/guidelines.xhtml

$$HCU_{ijl} = \beta.inflexible_{ijl} + \boldsymbol{\gamma_1 X_{ij}} + \tau_l + \delta_t + \varepsilon_{ijl} \quad (3)$$

$$H_{ijl} = \beta.\widehat{HCU}_{ijl} + \boldsymbol{\gamma_1 X_{ij}} + \tau_l + \delta_t + u_{ijl} \quad (4)$$

Two-stage least squares where equation (3) is the first stage and (4) is the second stage.

Standard Occupational Classifications (SOCs)

At the LAD level, we obtained annual information on the share of the working population employed in each of the nine broad categories of Standard Occupational Classification (SOC)[29]: 1) managers, directors and senior officials; 2) professional; 3) associate professional and technical; 4) administrative and secretarial; 5) skilled trade; 6) caring, leisure and other service; 7) sales and customer services; 8) process, plant and machine operatives; and 9) elementary occupations. We obtained these variables via NOMIS: the UK Labour Market statistics portal [30] and merged them by LAD to the individual-level data.

We intended to include the percentage of the population employed in each sector as a covariate to adjust for other job characteristics. However when included, they were not jointly statistically significant, and their inclusion did not alter the coefficient on the inflexible job indicator.

For peer review only

Supplementary Table 1: Possible responses to survey questions “when did you last see or speak to a GP [nurse] from your GP surgery?” and corresponding intervals in main analysis; and value assigned in two-stage least squares regressions

Response	Months since last saw/spoke to GP/nurse	Value in two-stage least squares regressions
In the past 3 months	0 to 3	1
Between 3 and 6 months ago	3 to 6	2
Between 6 and 12 months ago	6 to 12	3
More than 12 months ago	12 to 24	4
I have never seen a GP from my GP surgery	24+	5

Data source: General Practice Patient Survey, Questions 1 and 2

For peer review only

For peer review only - http://bmjopen.bmj.com/site/about/guidelines.xhtml

Supplementary Table 2: Local Authority Districts with lowest percentage of respondents reporting an inflexible job

Local Authority District	% reporting inflexible job	N
Isles of Scilly	13.1	175
City of London	19.2	240
Cambridge	19.4	2,847
Ryedale	20.7	1,282
Oxford	21.0	4,000
Melton	21.0	932
South Lakeland	21.3	2,987
South Cambridgeshire	21.8	4,509
West Somerset	21.9	987
Vale of White Horse	22.1	2,676
Cotswold	22.3	2,312
Rutland	22.4	706
Wokingham	22.8	3,156
South Oxfordshire	22.9	3,039
South Somerset	22.9	4,517
Stroud	22.9	3,377
Derbyshire Dales	23.1	1,969
West Oxfordshire	23.2	2,498
Wiltshire	23.3	10,662
Eden	23.3	1,813

Data source: GPPS respondents in full-time employment, 2012-2017

For peer review only

Supplementary Table 3: Local Authority Districts with highest percentage of respondents reporting an inflexible job

Local Authority District	% reporting inflexible job	N
Barking and Dagenham	40.7	6,772
Newham	38.3	9,043
Knowsley	37.8	4,237
Thurrock	37.6	5,690
Barnsley	37.4	5,560
Hillingdon	36.8	9,755
Basildon	36.8	5,752
Dartford	36.6	2,888
Havering	36.5	8,012
Luton	36.5	5,089
Sandwell	36.5	9,165
St. Helens	36.4	5,505
Bolsover	36.4	73
Medway	36.3	9,291
Mansfield	36.2	2,273
Crawley	36.1	2,499
Gravesham	36.1	2,506
Rotherham	36.1	5,677
Wakefield	36.1	6,635
Redcar and Cleveland	35.9	3,176

Data source: GPPS respondents in full-time employment, 2012-2017

For peer review only

Supplementary Table 4: Descriptive statistics: Percentage of respondents who gave each response for each EQ5D domain

	Mobility		Self-care		Usual activities		Pain/discomfort		Anxiety/ depression	
	Female	Male	Female	Male	Female	Male	Female	Male	Female	Male
1. No problems	91.16	90.47	98.44	98.35	87.74	88.02	63.34	63.81	71.17	77.21
2. Slight problems	6.49	6.98	1.09	1.15	9.27	8.99	26.35	27.15	20.00	15.98
3. Moderate problems	1.80	1.95	0.35	0.34	2.37	2.35	8.40	7.51	7.31	5.63
4. Severe problems	0.43	0.44	0.06	0.06	0.45	0.46	1.69	1.32	1.19	0.89
5. Unable to/extreme problems	0.12	0.16	0.06	0.09	0.18	0.19	0.23	0.20	0.33	0.27

Data source: GPPS respondents in full-time employment, 2012-2017

For peer review only

For peer review only - http://bmjopen.bmj.com/site/about/guidelines.xhtml

Supplementary Table 5: Descriptive statistics: Prevalence of long-term conditions (%)

	**Female**	**Male**
Alzheimers disease or dementia	0.02	0.04
Angina or long-term heart problem	0.57	1.64
Arthritis or long-term joint problem	4.37	3.74
Asthma or long-term chest problem	8.85	7.83
Blindness or severe visual impairment	0.12	0.21
Cancer in the last 5 years	1.37	0.99
Deafness or severe hearing impairment	0.72	1.11
Diabetes	2.21	4.22
Epilepsy	0.54	0.59
High blood pressure	6.14	9.67
Kidney or liver disease	0.57	0.78
Learning difficulty	0.09	0.17
Long-term back problem	5.12	5.74
Long-term mental health problem	3.29	1.97
Long-term neurological problem	0.89	0.67
Another long-term condition	10.14	8.01
Prefer not to say	1.77	1.91
**N**	570,626	662,258

Data source: GPPS respondents in full-time employment, 2012-2017

For peer review only

Supplementary Table 6: The effect of covariates on reporting an inflexible job and the effect of an inflexible job on the EQ-5D utility score; full sample of male and female, with interaction effects

	Inflexible job (1)	EQ-5D (2)
Inflexible job		-0.009
		[-0.012; -0.006]
Female	0.060	-0.010
	[0.058; 0.062]	[-0.011; -0.010]
Female * inflexible job		-0.002
		[-0.004; -0.001]
Parent	-0.001	0.013
	[-0.003; 0.002]	[0.012; 0.014]
Aged 25-34 years [a]	-0.063	-0.009
	[-0.068; -0.057]	[-0.011; -0.007]
Aged 35-44 years [a]	-0.093	-0.028
	[-0.098; -0.088]	[-0.029; -0.026]
Aged 45-54 years [a]	-0.104	-0.045
	[-0.109; -0.099]	[-0.046; -0.043]
Aged 55-64 years [a]	-0.133	-0.058
	[-0.138; -0.128]	[-0.060; -0.057]
Aged 25-34 years * inflexible job		-0.000
		[-0.003; 0.003]
Aged 35-44 years * inflexible job		-0.003
		[-0.006; -0.000]
Aged 45-54 years * inflexible job		-0.007
		[-0.010; -0.004]
Aged 55-64 years * inflexible job		-0.010
		[-0.013; -0.007]
Ethnicity: mixed [b]	0.001	-0.007
	[-0.009; 0.012]	[-0.010; -0.004]
Ethnicity: Asian [b]	0.018	0.007
	[0.014; 0.023]	[0.005; 0.008]
Ethnicity: black [b]	-0.020	0.010
	[-0.026; -0.013]	[0.008; 0.012]
Ethnicity: other [b]	0.011	-0.006
	[0.003; 0.019]	[-0.009; -0.003]
Never smoked	-0.011	0.026
	[-0.013; -0.008]	[0.025; 0.026]
Deprivation decile 2 [c]	0.006	-0.003
(least deprived)	[0.001; 0.010]	[-0.005; -0.002]
Deprivation decile 3 [c]	0.008	-0.006
	[0.003; 0.012]	[-0.008; -0.005]
Deprivation decile 4 [c]	0.018	-0.008
	[0.013; 0.022]	[-0.009; -0.006]
Deprivation decile 5 [c]	0.022	-0.009

For peer review only - http://bmjopen.bmj.com/site/about/guidelines.xhtml

	[0.017; 0.027]	[-0.011; -0.008]
Deprivation decile 6 [c]	0.028	-0.014
	[0.023; 0.033]	[-0.015; -0.012]
Deprivation decile 7 [c]	0.036	-0.017
	[0.031; 0.041]	[-0.019; -0.016]
Deprivation decile 8 [c]	0.043	-0.020
	[0.038; 0.048]	[-0.022; -0.018]
Deprivation decile 9 [c]	0.059	-0.022
	[0.053; 0.064]	[-0.024; -0.021]
Deprivation decile 10 [c]	0.072	-0.028
(most deprived)	[0.066; 0.077]	[-0.030; -0.026]
Deprivation decile 2 * inflexible job		0.001
(least deprived)		[-0.002; 0.004]
Deprivation decile 3 * inflexible job		0.003
		[-0.000; 0.006]
Deprivation decile 4 * inflexible job		0.000
		[-0.003; 0.003]
Deprivation decile 5 * inflexible job		-0.003
		[-0.006; -0.000]
Deprivation decile 6 * inflexible job		-0.002
		[-0.005; 0.001]
Deprivation decile 7 * inflexible job		-0.005
		[-0.008; -0.002]
Deprivation decile 8 * inflexible job		-0.004
		[-0.007; -0.001]
Deprivation decile 9 * inflexible job		-0.005
		[-0.008; -0.002]
Deprivation decile 10 * inflexible job		-0.006
(most deprived)		[-0.010; -0.003]
N	1,232,884	1,232,884

Data source: GPPS pooled data for 2012 to 2017.
95% confidence intervals in parentheses.
Models estimated using OLS.
[a] The reference category for age group is 16-24 years;
[b] The reference category for ethnic group is white;
[c] The reference category for deprivation is decile 1;
Models additionally include fixed effects for survey year and Local Authority District of residence.

For peer review only

Supplementary Table 7: The effect of covariates on reporting an inflexible job and the effect of an inflexible job on the EQ-5D utility score; full-time and part-time workers

	**Inflexible job**		**EQ-5D**	
	Females	Males	Females	Males
	(1)	(2)	(3)	(4)
Inflexible job			-0.018	-0.016
			[-0.018; -0.017]	[-0.017; -0.015]
Part-time	0.027	-0.017	-0.016	-0.039
	[0.025; 0.030]	[-0.021; -0.012]	[-0.017; -0.015]	[-0.040; -0.037]
Parent	-0.005	0.002	0.025	0.012
	[-0.008; -0.002]	[-0.002; 0.005]	[0.024; 0.025]	[0.011; 0.013]
Aged 25-34 years [a]	-0.054	-0.060	-0.000	-0.023
	[-0.060; -0.048]	[-0.067; -0.052]	[-0.002; 0.001]	[-0.025; -0.021]
Aged 35-44 years [a]	-0.076	-0.088	-0.020	-0.042
	[-0.082; -0.070]	[-0.095; -0.080]	[-0.022; -0.018]	[-0.044; -0.040]
Aged 45-54 years [a]	-0.086	-0.100	-0.039	-0.061
	[-0.092; -0.080]	[-0.107; -0.093]	[-0.040; -0.037]	[-0.062; -0.059]
Aged 55-64 years [a]	-0.117	-0.131	-0.047	-0.073
	[-0.123; -0.111]	[-0.138; -0.124]	[-0.049; -0.045]	[-0.074; -0.071]
Ethnicity: mixed [b]	0.001	0.006	-0.014	-0.006
	[-0.010; 0.013]	[-0.008; 0.020]	[-0.017; -0.010]	[-0.011; -0.002]
Ethnicity: Asian [b]	-0.002	0.017	0.001	0.008
	[-0.007; 0.004]	[0.011; 0.022]	[-0.001; 0.003]	[0.007; 0.010]
Ethnicity: black [b]	-0.037	-0.010	-0.002	0.020
	[-0.045; -0.030]	[-0.019; -0.002]	[-0.004; 0.000]	[0.017; 0.022]
Ethnicity: other [b]	-0.016	0.007	-0.013	-0.004
	[-0.025; -0.006]	[-0.002; 0.015]	[-0.017; -0.010]	[-0.007; -0.000]
Never smoked	0.009	-0.022	0.028	0.026
	[0.007; 0.012]	[-0.025; -0.019]	[0.027; 0.028]	[0.025; 0.027]
Deprivation decile 1 [c]	-0.005	-0.029	0.012	0.012
(least deprived)	[-0.011; 0.001]	[-0.035; -0.023]	[0.010; 0.014]	[0.010; 0.014]
Deprivation decile 2 [c]	-0.009	-0.019	0.007	0.008
	[-0.014; -0.003]	[-0.025; -0.013]	[0.006; 0.009]	[0.006; 0.010]
Deprivation decile 3 [c]	-0.007	-0.018	0.005	0.006
	[-0.012; -0.001]	[-0.024; -0.012]	[0.004; 0.007]	[0.004; 0.008]
Deprivation decile 4 [c]	-0.005	-0.005	0.002	0.003
	[-0.010; 0.000]	[-0.012; 0.001]	[0.000; 0.004]	[0.001; 0.005]
Deprivation decile 6 [c]	0.007	0.007	-0.005	-0.003
	[0.002; 0.013]	[0.001; 0.013]	[-0.007; -0.004]	[-0.005; -0.002]
Deprivation decile 7 [c]	0.009	0.016	-0.010	-0.007
	[0.004; 0.015]	[0.010; 0.022]	[-0.012; -0.008]	[-0.009; -0.006]
Deprivation decile 8 [c]	0.014	0.024	-0.015	-0.010
	[0.009; 0.020]	[0.017; 0.030]	[-0.016; -0.013]	[-0.012; -0.008]
Deprivation decile 9 [c]	0.026	0.039	-0.018	-0.013

For peer review only

	[0.020; 0.032]	[0.032; 0.045]	[-0.020; -0.017]	[-0.015; -0.010]
Deprivation decile 10 [c]	0.037	0.049	-0.025	-0.017
(most deprived)	[0.030; 0.043]	[0.042; 0.056]	[-0.027; -0.024]	[-0.020; -0.015]
N	912,598	742,534	912,598	742,534

Data source: GPPS pooled data for 2012 to 2017.
95% confidence intervals in parentheses.
Models estimated using OLS.
[a] The reference category for age group is 16-24 years;
[b] The reference category for ethnic group is white;
[c] The reference category for deprivation is decile 5;
Models additionally include fixed effects for survey year and Local Authority District of residence.

For peer review only

For peer review only - http://bmjopen.bmj.com/site/about/guidelines.xhtml

Supplementary Table 8: The effect of an inflexible job on healthcare utilisation

	Months since last saw GP		Months since last saw nurse	
	Females (1)	Males (2)	Females (3)	Males (4)
Inflexible job	0.234	0.199	0.569	0.653
	[0.201; 0.268]	[0.152; 0.247]	[0.519; 0.618]	[0.586; 0.720]
Parent	-1.341	-0.926	-1.397	-0.307
	[-1.377; -1.305]	[-0.975; -0.877]	[-1.450; -1.343]	[-0.376; -0.239]
Aged 25-34 years [a]	0.202	-0.053	-1.097	-0.251
	[0.132; 0.272]	[-0.175; 0.069]	[-1.208; -0.986]	[-0.423; -0.080]
Aged 35-44 years [a]	0.830	-0.357	-0.206	-1.130
	[0.759; 0.901]	[-0.475; -0.239]	[-0.317; -0.094]	[-1.297; -0.963]
Aged 45-54 years [a]	1.055	-0.571	0.497	-2.088
	[0.988; 1.122]	[-0.684; -0.457]	[0.391; 0.604]	[-2.248; -1.928]
Aged 55-64 years [a]	1.233	-1.031	0.933	-2.990
	[1.161; 1.305]	[-1.145; -0.918]	[0.820; 1.045]	[-3.151; -2.829]
Ethnicity: mixed [b]	-0.135	-0.325	-0.205	-0.521
	[-0.273; 0.002]	[-0.550; -0.100]	[-0.418; 0.008]	[-0.853; -0.189]
Ethnicity: Asian [b]	-0.034	-1.151	0.420	-1.523
	[-0.108; 0.040]	[-1.237; -1.065]	[0.304; 0.537]	[-1.649; -1.397]
Ethnicity: black [b]	-0.492	-1.133	-0.436	-2.228
	[-0.573; -0.410]	[-1.255; -1.010]	[-0.572; -0.300]	[-2.412; -2.044]
Ethnicity: other [b]	-0.035	-0.888	-0.167	-1.947
	[-0.173; 0.103]	[-1.035; -0.742]	[-0.366; 0.032]	[-2.157; -1.737]
Never smoked	0.402	0.359	0.329	0.305
	[0.370; 0.435]	[0.317; 0.402]	[0.281; 0.378]	[0.245; 0.364]
Deprivation decile 1 [c]	-0.014	0.030	0.017	0.154
(least deprived)	[-0.087; 0.058]	[-0.066; 0.126]	[-0.091; 0.124]	[0.021; 0.287]
Deprivation decile 2 [c]	-0.038	0.001	0.024	0.086
	[-0.107; 0.031]	[-0.092; 0.095]	[-0.080; 0.128]	[-0.044; 0.216]
Deprivation decile 3 [c]	0.022	-0.034	0.082	0.093
	[-0.048; 0.092]	[-0.128; 0.060]	[-0.021; 0.185]	[-0.037; 0.222]
Deprivation decile 4 [c]	-0.044	-0.023	-0.025	0.034
	[-0.113; 0.025]	[-0.117; 0.070]	[-0.127; 0.077]	[-0.096; 0.164]
Deprivation decile 6 [c]	0.029	-0.028	0.067	0.001
	[-0.041; 0.098]	[-0.124; 0.067]	[-0.037; 0.170]	[-0.132; 0.133]
Deprivation decile 7 [c]	-0.093	-0.013	0.005	0.030
	[-0.163; -0.023]	[-0.109; 0.084]	[-0.099; 0.110]	[-0.105; 0.165]
Deprivation decile 8 [c]	-0.040	0.033	0.033	-0.045
	[-0.112; 0.031]	[-0.065; 0.131]	[-0.073; 0.140]	[-0.181; 0.092]
Deprivation decile 9 [c]	-0.003	-0.031	-0.062	-0.084
	[-0.077; 0.070]	[-0.132; 0.070]	[-0.171; 0.047]	[-0.224; 0.057]
Deprivation decile 10 [c]	-0.062	-0.070	0.028	-0.124
(most deprived)	[-0.140; 0.015]	[-0.174; 0.034]	[-0.087; 0.143]	[-0.269; 0.021]

For peer review only - http://bmjopen.bmj.com/site/about/guidelines.xhtml

For peer review only

Appointment availability	-2.756	-3.262	0.338	1.374
	[-3.077; -2.436]	[-3.643; -2.880]	[-0.099; 0.774]	[0.850; 1.899]
Alzheimers	1.320	2.788	0.650	2.558
	[0.034; 2.607]	[1.134; 4.442]	[-1.147; 2.447]	[0.246; 4.871]
Angina	-0.944	-2.177	-1.391	-3.777
	[-1.109; -0.779]	[-2.282; -2.073]	[-1.632; -1.149]	[-3.935; -3.619]
Arthritis	-1.247	-1.707	-0.925	-1.254
	[-1.302; -1.192]	[-1.783; -1.631]	[-1.018; -0.831]	[-1.378; -1.131]
Asthma	-1.098	-1.955	-2.312	-4.488
	[-1.144; -1.053]	[-2.022; -1.888]	[-2.381; -2.243]	[-4.584; -4.392]
Blindness	-0.004	0.587	1.281	1.035
	[-0.387; 0.380]	[0.104; 1.070]	[0.494; 2.068]	[0.351; 1.719]
Cancer in past 5 years	-1.361	-2.347	-1.050	-2.956
	[-1.460; -1.263]	[-2.487; -2.207]	[-1.212; -0.888]	[-3.189; -2.723]
Deafness	-0.380	-0.537	-0.290	-0.250
	[-0.538; -0.222]	[-0.693; -0.380]	[-0.540; -0.039]	[-0.488; -0.011]
Diabetes	-1.387	-2.579	-3.767	-6.546
	[-1.460; -1.315]	[-2.644; -2.515]	[-3.863; -3.670]	[-6.626; -6.465]
Epilepsy	-1.253	-2.297	-0.510	-1.799
	[-1.404; -1.102]	[-2.523; -2.072]	[-0.828; -0.193]	[-2.211; -1.387]
High blood pressure	-1.295	-2.339	-2.486	-4.421
	[-1.344; -1.247]	[-2.387; -2.292]	[-2.558; -2.415]	[-4.489; -4.352]
Kidney/liver disease	-0.819	-1.435	-1.200	-2.038
	[-0.984; -0.655]	[-1.612; -1.258]	[-1.446; -0.955]	[-2.331; -1.744]
Learning difficulties	0.677	0.352	0.648	-0.243
	[-0.038; 1.392]	[-0.261; 0.964]	[-0.421; 1.717]	[-1.086; 0.600]
Back problem	-0.896	-1.368	-0.134	-0.511
	[-0.953; -0.839]	[-1.439; -1.297]	[-0.233; -0.034]	[-0.627; -0.395]
Mental health	-1.864	-3.461	-0.231	-1.379
	[-1.922; -1.806]	[-3.566; -3.355]	[-0.362; -0.101]	[-1.599; -1.160]
Neurological	-1.405	-2.161	-0.832	-1.745
	[-1.515; -1.295]	[-2.350; -1.972]	[-1.047; -0.617]	[-2.079; -1.411]
Another condition	-1.574	-2.569	-1.445	-2.638
	[-1.613; -1.534]	[-2.627; -2.512]	[-1.514; -1.376]	[-2.735; -2.540]
Prefer not to say	-1.622	-1.927	-0.607	-1.734
	[-1.724; -1.521]	[-2.081; -1.773]	[-0.796; -0.419]	[-1.968; -1.499]
N	570,626	662,258	570,626	662,258

Data source: GPPS pooled data for 2012 to 2017.
95% confidence intervals in parentheses.
Models estimated using interval regression.
[a] The reference category for age group is 16-24 years;
[b] The reference category for ethnic group is white;
[c] The reference category for deprivation is decile 5;
Models additionally include fixed effects for survey year and Local Authority District of residence.

For peer review only - http://bmjopen.bmj.com/site/about/guidelines.xhtml

Supplementary Table 9: Sensitivity analyses: altering the intervals in health care use regressions

Survey response category	Intervals (months since last seen GP/nurse)			Extensive margin: never seen GP/nurse before = 1
	**Main analysis**	**Sensitivity 1**	**Sensitivity 2**	**Sensitivity 3**
In the past 3 months	0 to 3	0 to 3	0 to 3	
Between 3 and 6 months ago	3 to 6	3 to 6	3 to 6	
Between 6 and 12 months ago	6 to 12	6 to 12	6 to 12	Have seen before
More than 12 months ago	12 to 24	12 to 60	12+	
I have never seen a GP/nurse from my GP surgery	24+	60+		Never seen before

General Practice Patient Survey, Questions 1 and 2

For peer review only

Supplementary Table 10: Results of sensitivity analyses altering the intervals in health care use regressions: coefficient on inflexible job

	Months since last saw GP		Months since last saw nurse	
	Female (1)	Males (2)	Females (3)	Males (4)
**Main analysis**				
Inflexible job	0.234	0.199	0.569	0.653
	[0.201; 0.268]	[0.152; 0.247]	[0.519; 0.618]	[0.586; 0.720]
**Sensitivity 1**				
Inflexible job	0.288	0.316	1.056	1.783
	[0.232; 0.344]	[0.222; 0.409]	[0.947; 1.165]	[1.600; 1.970]
**Sensitivity 2**				
Inflexible job	0.221	0.180	0.486	0.459
	[0.192; 0.251]	[0.138; 0.221]	[0.445; 0.527]	[0.402; 0.516]
**Sensitivity 3**				
Inflexible job	0.001	0.001	0.006	0.021
	[0.0001; 0.002]	[0.0002; 0.003]	[0.005; 0.008]	[0.018; 0.023]
N	570,626	662,258	570,626	662,258

Data source: GPPS pooled data for 2012 to 2017.
95% confidence intervals in parentheses.
All models additionally include indicators for: parental status, ten-year age group, ethnicity, whether the respondent has ever smoked, index of multiple deprivation decile, 16 long-term conditions, practice level appointment availability, and fixed effects for survey year and Local Authority District of residence.
Main analysis, Sensitivity 1 and Sensitivity 2 estimated using interval regression.
Sensitivity 3 models (1), (3) and (4) estimated using logistic regression, results show marginal effects.
Sensitivity 3 model (2) estimated using OLS due to non-convergence of logit model.

For peer review only

Supplementary Table 11: The effect of an inflexible job on healthcare utilisation: out-of hours

	Females (1)	Males (2)
Inflexible job	0.197	0.234
	[0.157,0.238]	[0.184,0.284]
Parent	0.313	0.306
	[0.266,0.361]	[0.253,0.359]
Aged 25-34 years [a]	-0.352	-0.215
	[-0.422,-0.282]	[-0.325,-0.105]
Aged 35-44 years [a]	-0.768	-0.497
	[-0.843,-0.694]	[-0.606,-0.387]
Aged 45-54 years [a]	-0.977	-0.724
	[-1.048,-0.906]	[-0.831,-0.617]
Aged 55-64 years [a]	-1.074	-0.83
	[-1.157,-0.990]	[-0.942,-0.717]
Ethnicity: mixed [b]	0.081	0.024
	[-0.095,0.258]	[-0.213,0.261]
Ethnicity: Asian [b]	0.241	0.347
	[0.153,0.328]	[0.262,0.432]
Ethnicity: black [b]	0.074	-0.033
	[-0.047,0.194]	[-0.194,0.129]
Ethnicity: other [b]	0.395	0.66
	[0.265,0.525]	[0.547,0.773]
Never smoked	-0.126	-0.068
	[-0.167,-0.084]	[-0.116,-0.020]
Deprivation decile 1 [c]	-0.009	-0.089
(least deprived)	[-0.107,0.090]	[-0.205,0.026]
Deprivation decile 2 [c]	0.046	-0.142
	[-0.049,0.141]	[-0.257,-0.028]
Deprivation decile 3 [c]	-0.008	0.016
	[-0.101,0.085]	[-0.095,0.127]
Deprivation decile 4 [c]	0.067	0.028
	[-0.025,0.159]	[-0.081,0.137]
Deprivation decile 6 [c]	0.097	0.035
	[0.006,0.189]	[-0.077,0.147]
Deprivation decile 7 [c]	0.074	0.081
	[-0.017,0.166]	[-0.029,0.191]
Deprivation decile 8 [c]	0.036	0.123
	[-0.058,0.129]	[0.008,0.237]
Deprivation decile 9 [c]	0.108	0.189
	[0.013,0.204]	[0.075,0.303]
Deprivation decile 10 [c]	0.148	0.272
(most deprived)	[0.050,0.245]	[0.158,0.385]
Appointment availability	-0.898	-1.088
	[-1.241,-0.556]	[-1.479,-0.697]

For peer review only

Alzheimers	1.35	2.12
	[0.410,2.289]	[1.457,2.783]
Angina	0.497	0.584
	[0.306,0.689]	[0.447,0.722]
Arthritis	0.213	0.198
	[0.125,0.301]	[0.095,0.301]
Asthma	0.505	0.4
	[0.445,0.565]	[0.323,0.476]
Blindness	-0.262	0.035
	[-0.883,0.359]	[-0.520,0.590]
Cancer in past 5 years	0.481	0.441
	[0.338,0.623]	[0.258,0.624]
Deafness	0.27	0.036
	[0.059,0.480]	[-0.162,0.234]
Diabetes	0.446	0.387
	[0.330,0.561]	[0.295,0.478]
Epilepsy	0.233	0.272
	[-0.003,0.469]	[0.002,0.542]
High blood pressure	0.107	0.152
	[0.026,0.187]	[0.079,0.225]
Kidney/liver disease	0.471	0.629
	[0.270,0.672]	[0.437,0.822]
Learning difficulties	0.116	0.34
	[-0.284,0.516]	[-0.004,0.684]
Back problem	0.25	0.472
	[0.171,0.330]	[0.388,0.557]
Mental health	0.405	0.569
	[0.305,0.504]	[0.418,0.720]
Neurological	0.657	0.424
	[0.488,0.826]	[0.202,0.647]
Another condition	0.511	0.522
	[0.455,0.568]	[0.451,0.593]
Prefer not to say	0.445	0.507
	[0.309,0.582]	[0.357,0.657]
N	293,266	336,890

Data source: GPPS pooled data for 2012 to 2014.
95% confidence intervals in parentheses.
Models estimated using logistic regression.
[a] The reference category for age group is 16-24 years;
[b] The reference category for ethnic group is white;
[c] The reference category for deprivation is decile 5;
Models additionally include fixed effects for survey year and Local Authority District of residence.

For peer review only

Supplementary Table 12: Two-stage least squares regression results: the mediating effect of nurse visits in the effect of an inflexible job on health

	First stage: Time since last saw nurse		Second stage: EQ-5D utility score	
	Females	Males	Females	Males
	(1)	(2)	(3)	(4)
Time since last saw nurse			-0.175	-0.207
			[-0.195,-0.155]	[-0.238,-0.177]
Inflexible job	0.095	0.076		
	[0.086,0.103]	[0.066,0.085]		
Parent	-0.232	-0.019	-0.023	0.007
	[-0.242,-0.222]	[-0.029,-0.010]	[-0.028,-0.018]	[0.004,0.009]
Aged 25-34 years [a]	-0.174	-0.053	-0.029	-0.030
	[-0.193,-0.155]	[-0.075,-0.030]	[-0.034,-0.024]	[-0.036,-0.024]
Aged 35-44 years [a]	-0.022	-0.223	-0.023	-0.084
	[-0.041,-0.003]	[-0.245,-0.201]	[-0.027,-0.019]	[-0.093,-0.075]
Aged 45-54 years [a]	0.066	-0.454	-0.026	-0.150
	[0.048,0.084]	[-0.475,-0.433]	[-0.030,-0.023]	[-0.165,-0.135]
Aged 55-64 years [a]	0.056	-0.767	-0.040	-0.230
	[0.037,0.075]	[-0.789,-0.746]	[-0.044,-0.036]	[-0.254,-0.206]
Ethnicity: mixed [b]	-0.055	-0.098	-0.019	-0.025
	[-0.092,-0.017]	[-0.145,-0.051]	[-0.028,-0.011]	[-0.036,-0.013]
Ethnicity: Asian [b]	0.049	-0.278	0.013	-0.050
	[0.030,0.069]	[-0.296,-0.260]	[0.009,0.018]	[-0.059,-0.041]
Ethnicity: black [b]	-0.111	-0.336	-0.019	-0.052
	[-0.135,-0.088]	[-0.363,-0.308]	[-0.024,-0.013]	[-0.064,-0.040]
Ethnicity: other [b]	-0.077	-0.334	-0.023	-0.074
	[-0.111,-0.043]	[-0.365,-0.304]	[-0.031,-0.016]	[-0.086,-0.061]
Never smoked	0.069	0.072	0.038	0.040
	[0.060,0.077]	[0.064,0.081]	[0.036,0.041]	[0.037,0.043]
Deprivation decile 1 [c]	0.023	0.053	0.013	0.022
(least deprived)	[0.004,0.042]	[0.034,0.072]	[0.009,0.017]	[0.017,0.027]
Deprivation decile 2 [c]	0.015	0.035	0.008	0.015
	[-0.004,0.033]	[0.016,0.054]	[0.004,0.012]	[0.011,0.020]
Deprivation decile 3 [c]	0.021	0.029	0.008	0.012
	[0.003,0.039]	[0.010,0.047]	[0.004,0.012]	[0.007,0.016]
Deprivation decile 4 [c]	-0.003	0.012	0.001	0.006
	[-0.021,0.016]	[-0.006,0.031]	[-0.003,0.005]	[0.001,0.010]
Deprivation decile 6 [c]	0.003	-0.003	-0.004	-0.004
	[-0.016,0.021]	[-0.022,0.016]	[-0.008,-0.000]	[-0.008,0.001]
Deprivation decile 7 [c]	-0.009	-0.014	-0.011	-0.011
	[-0.028,0.009]	[-0.034,0.005]	[-0.015,-0.007]	[-0.015,-0.006]
Deprivation decile 8 [c]	-0.010	-0.032	-0.014	-0.016
	[-0.029,0.009]	[-0.052,-0.012]	[-0.018,-0.010]	[-0.021,-0.012]
Deprivation decile 9 [c]	-0.032	-0.043	-0.021	-0.022

For peer review only

For peer review only - http://bmjopen.bmj.com/site/about/guidelines.xhtml

	[-0.051,-0.012]	[-0.063,-0.023]	[-0.026,-0.017]	[-0.027,-0.017]
Deprivation decile 10 [c]	-0.025	-0.073	-0.027	-0.033
(most deprived)	[-0.046,-0.005]	[-0.094,-0.052]	[-0.032,-0.023]	[-0.038,-0.027]
N	570,626	662,258	570,626	662,258

Data source: GPPS pooled data for 2012 to 2017.
95% confidence intervals in parentheses.
[a] The reference category for age group is 16-24 years.
[b] The reference category for ethnic group is white.
[c] The reference category for deprivation is decile 5.
Models additionally include fixed effects for survey year and Local Authority District of residence.

For peer review only

Supplementary Figure 1: Flowchart of observations dropped at each stage of data preparation

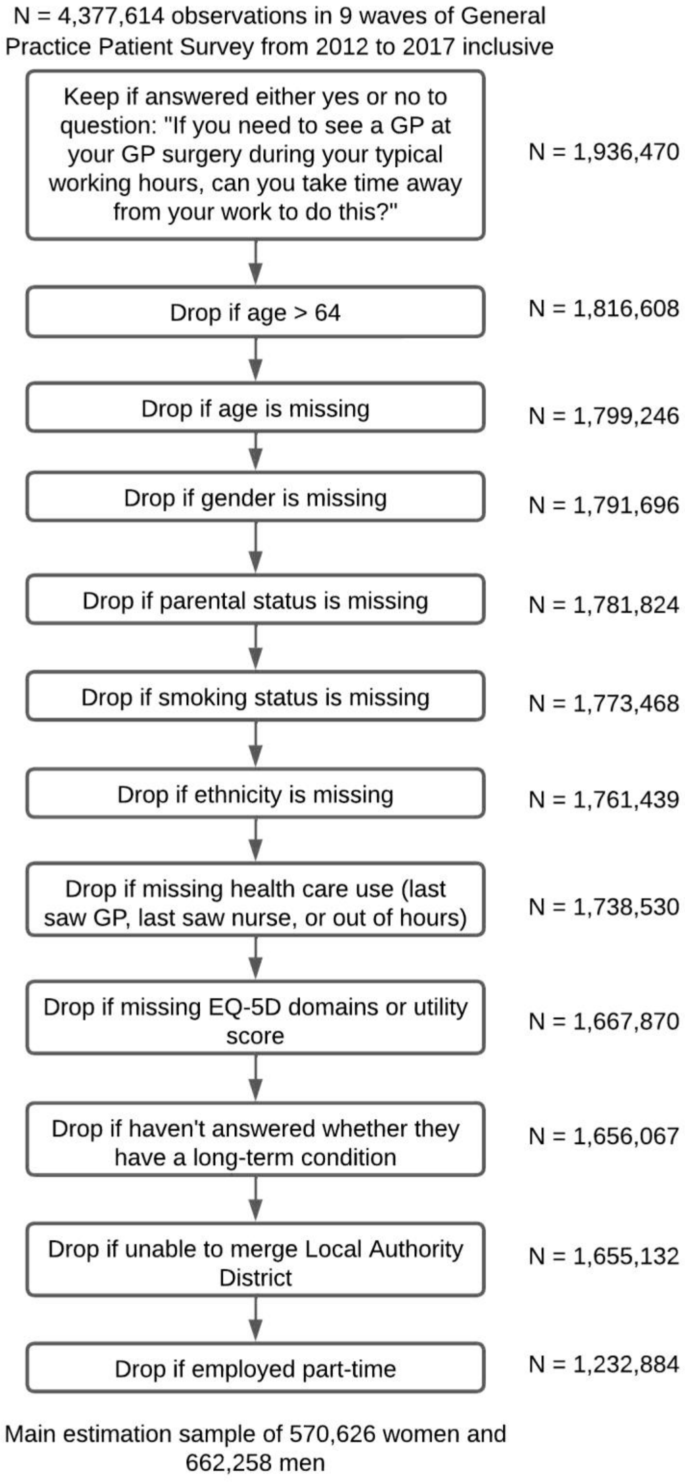

For peer review only - http://bmjopen.bmj.com/site/about/guidelines.xhtml

Supplementary Figure 2: Geographic distribution of inflexible jobs and health-related quality of life in England

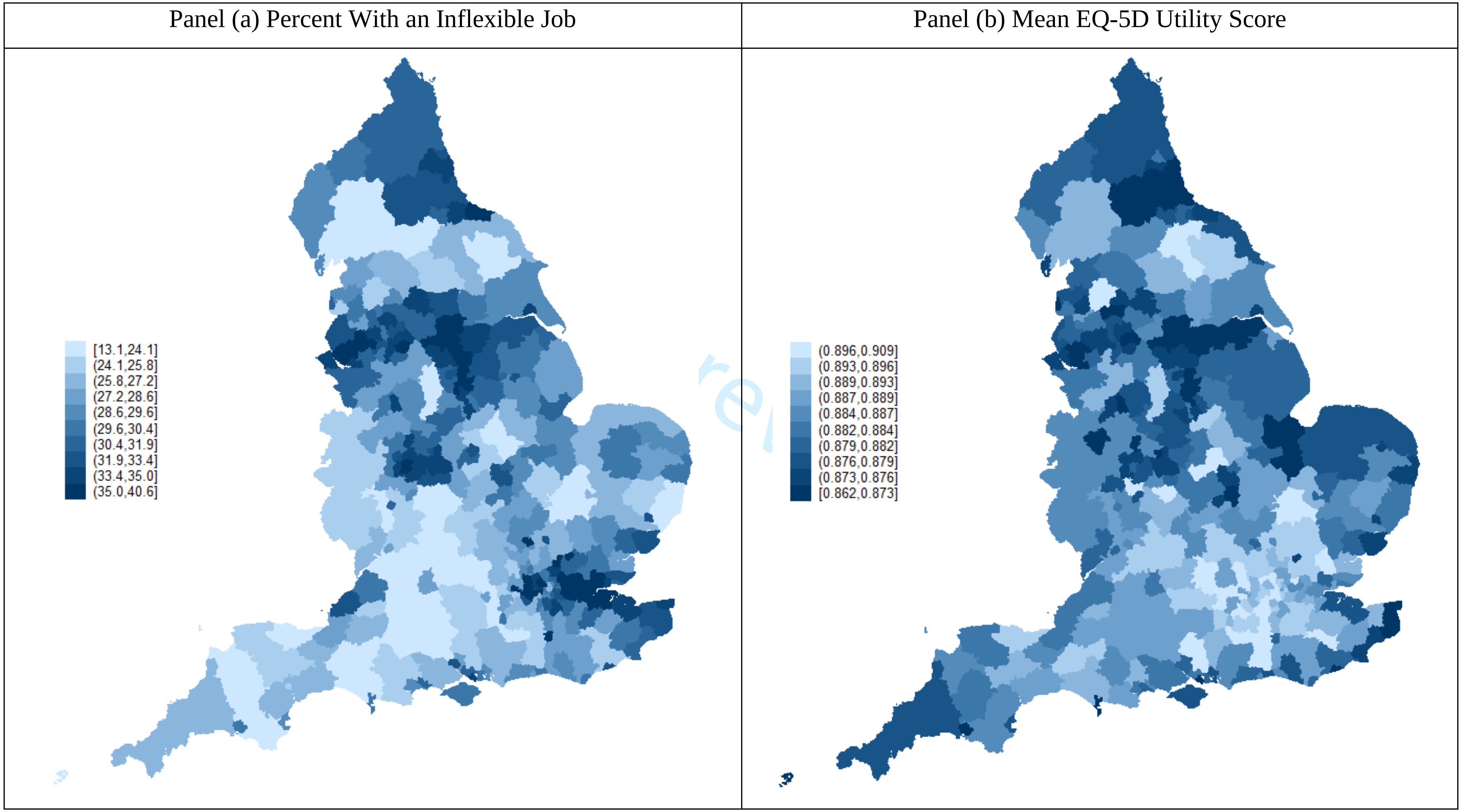

Data source: 1,346,001 General Practice Patient Survey respondents in full-time employment who have complete data for questions relating to job flexibility and EQ-5D utility score; pooled data from 2012-2017; respondents who report inability to visit their GP during working hours are classified as having an inflexible job; averages for 326 local authority districts; maps created using spmap (Pisati, 2018); deciles generated within spmap
